# High-Frequency Nanosecond Bleomycin Electrochemotherapy and its Effects on Changes in the Immune System and Survival

**DOI:** 10.3390/cancers14246254

**Published:** 2022-12-19

**Authors:** Austėja Balevičiūtė, Eivina Radzevičiūtė, Augustinas Želvys, Veronika Malyško-Ptašinskė, Jurij Novickij, Auksė Zinkevičienė, Vytautas Kašėta, Vitalij Novickij, Irutė Girkontaitė

**Affiliations:** 1 Institute of Environmental Medicine, Toxicology Unit, Karolinska Institutet, 171 77 Stockholm, Sweden; 2 Department of Immunology, State Research Institute Centre for Innovative Medicine, 13102 Vilnius, Lithuania; 3 Faculty of Electronics, Vilnius Gediminas Technical University, 10223 Vilnius, Lithuania; 4 Department of Biomodels, State Research Institute Centre for Innovative Medicine, 11342 Vilnius, Lithuania

**Keywords:** nanosecond, electroporation, electrochemotherapy, in vivo, bleomycin, pulsed electric fields, immunology, immunomodulation, anticancer, humoral and cellular immunity

## Abstract

**Simple Summary:**

Standard microsecond electrochemotherapy (μsECT) is used in clinical trials for the elimination of tumours, while the first studies on nanosecond electrochemotherapy (nsECT) only started to appear recently. Nanosecond pulses enable more homogeneous treatment and better control of pulse burst energy, and thus the whole field of electroporation is moving towards the shorter pulse range. In order to ensure a full anticancer response and potentially prevent any metastases, the immunomodulatory effects should also be induced. Therefore, in this work, we used nsECT protocols based on kHz and MHz pulse bursts and characterized the response of the immune system to the novel modality of nano-electrochemotherapy. The results of this study are useful for the development of effective anticancer treatment strategies based on high frequency nanosecond electric fields.

**Abstract:**

In this work, a time-dependent and time-independent study on bleomycin-based high-frequency nsECT (3.5 kV/cm × 200 pulses) for the elimination of LLC1 tumours in C57BL/6J mice is performed. We show the efficiency of nsECT (200 ns and 700 ns delivered at 1 kHz and 1 MHz) for the elimination of tumours in mice and increase of their survival. The dynamics of the immunomodulatory effects were observed after electrochemotherapy by investigating immune cell populations and antitumour antibodies at different timepoints after the treatment. ECT treatment resulted in an increased percentage of CD4^+^ T, splenic memory B and tumour-associated dendritic cell subsets. Moreover, increased levels of antitumour IgG antibodies after ECT treatment were detected. Based on the time-dependent study results, nsECT treatment upregulated PD 1 expression on splenic CD4^+^ Tr1 cells, increased the expansion of splenic CD8^+^ T, CD4^+^CD8^+^ T, plasma cells and the proportion of tumour-associated pro inflammatory macrophages. The Lin^−^ population of immune cells that was increased in the spleens and tumour after nsECT was identified. It was shown that nsECT prolonged survival of the treated mice and induced significant changes in the immune system, which shows a promising alliance of nanosecond electrochemotherapy and immunotherapy.

## 1. Introduction

Electroporation (EP) leads to the formation of nanoscale aqueous pores in the cell plasma membrane, facilitating the delivery of impermeable exogenous molecules [1,2]. In the biomedical context, electroporation is typically used for tissue ablation, electrochemotherapy (ECT), DNA and RNA vaccination or gene therapy [3,4,5]. Tissue ablation relies on irreversible damage caused by the high pulsed electric field (PEFs), while the electrochemotherapy and gene vaccination method require reversible permeabilization of cells accompanied by molecular transport to target molecules. As a result, the parametric protocols vary significantly in terms of electric field amplitude, pulse duration and number of pulses. Electrochemotherapy appears to be more standardized when compared to PEF-based tissue ablation methods—the dominating fraction of applied ECT works is based on the European Standard Operating Procedures of Electrochemotherapy (ESOPE) [6]. The protocol involves bursts of 8 × 100 μs pulses with amplitudes ranging from 1 kV/cm to 1.5 kV/cm for reversible permeabilization of cancer cells [7]. Nevertheless, in the past decade, many electroporation-based treatments move towards the shorter pulse duration range (nanosecond or short microsecond), while in the context of electrochemotherapy works based on nanosecond pulses (nsPEF), it started to appear only recently [8,9,10]. The motivation to use nsPEF in ECT is influenced by the higher frequency component of the pulse bursts and the PEF amplitudes far beyond the electroporation threshold, which is beneficial for the treatment of heterogeneous tumours. The short duration of the pulse nsPEF also enables a more precise management of Joule heating [11,12], minimization of muscle contractions [13] and products of electrolysis [14]. The lack of nsECT works can be explained by the technological challenges to generate high intensity nanosecond pulses and more importantly by the lack of significant electrophoretic force, which contributes to the successful intracellular delivery of cytotoxic drugs. In order to compensate for the lack of electro-diffusive force, a higher drug dose can be used to ensure a higher concentration gradient for a more rapid diffusion. However, increasing the drug dose increases the overall toxicity of the treatment to patients [15,16,17], therefore tuning of the electric field parameters is a better solution. Recent studies have shown that bleomycin, doxorubicin and cisplatin-based nsECTs can be as effective as μsECT [8,14,18,19,20].

Chemotherapeutics are crucial for ECT-based treatments, and bleomycin and cisplatin were introduced in preclinical trials several decades ago [21]. These antineoplastic drugs have been shown to be suitable for the efficient elimination of solid tumours [22,23,24]. Once inside the cancer cells, these drugs inhibit the activity of essential enzymes or disrupt DNA synthesis, inducing cell death. Bleomycin is still one of the most popular antineoplastic drugs [25] and has been in use since the 1970s. This chemotherapeutic drug is difficult to transport through the membranes of cancer cells via passive diffusion due to its high molecular weight. Electroporation is successfully used as a physical method to facilitate intracellular delivery. When inside, bleomycin interferes with the DNA strand and causes structural changes in the chromosome morphology that triggers various cell signal-transduction cascades that lead to different fates (cell-cycle arrest, DNA repair, mitotic cell death, necroptosis, apoptosis or other) [26,27]. Cisplatin is another widely used drug that has shown anticancer activity in a variety of tumours, including cancers of the ovaries and solid tumours of the head and neck. Cisplatin belongs to a class of alkylating agents that directly attack DNA by cross-linking guanine bases, causing disruption of DNA function and inducing cell death [28]. However, doxorubicin is an antibiotic, belonging to the anthracycline group. Although, six decades ago, it was repurposed as a chemotherapeutic agent used for the treatment of various types of cancers. Doxorubicin disrupts the DNA repair machinery by intercalating DNA bases and inducing the generation of free radicals, further damaging cellular compartments (membranes, DNA, proteins) [29]. Therefore, these drugs must be administered at high toxic doses that result in adverse side effects [30,31]. This study is limited to bleomycin-based nsECT due to the dominant popularity of this drug in the electrochemotherapy context.

Whether alone, combined with chemotherapeutics, or other treatment modalities, the effects of electric pulses on changes in the immune system are still poorly explored, while the promising alliance of electroporation-based treatment was predicted [7,32,33,34]. It was shown that only electroporation of tumours can induce T cell activation and immune memory. Other than adaptive immune response, electroporation can also promote macrophage (MØ) polarization towards the pro-inflammatory phenotype. It is thought that electroporation can release tumour cell antigens and stimulate innate and adaptive immune responses. Changes in immune cells after electroporation can promote antitumour immunity or delay the onset of rapid tumour progression [35,36,37]. Electroporation, combined with chemotherapeutics, induces the release of ATP in affected tumour cells. Induced immunological cell death (ICD) occurs when calreticulin is exposed to the cell membrane surface and the high mobility group box 1 (HMGB1) protein is released to the extracellular space, further activating the production and release of pro-inflammatory cytokines [38]. Bleomycin ECT induces inhibition of angiogenesis in tumours by completely stopping the tumour blood vessels for up to 24 h after the treatment. Furthermore, no damage to peritumoral normal blood vessels was induced [39,40]. Immune system-wise, bleomycin ECT increases the infiltration of CD8^+^ T lymphocytes into the tumours by recruiting dendritic cells (DCs), while the presence of CD4^+^ T cells remains stable [41,42]. Similarly to bleomycin ECT, cisplatin ECT also increases the ratio of tumour-infiltrating CD8^+^ T cells. Furthermore, cisplatin ECT positively affects the recruitment of macrophages, neutrophils, B, NK, natural killer T cells, and DCs to the tumour microenvironment [43,44]. Cisplatin ECT was shown to delay tumour growth in immunodeficient mice for twice as long as in immunocompetent mice [45]. Application of nanosecond-duration pulses for cancer treatment also shows positive effects on the immune system. Orthotopic hepatocellular carcinoma (HCC) tumours were successfully ablated with nsPEF in a rat model and newly inoculated tumours failed to re-grow. Additionally, it was observed that, within days after the treatment, tumours are infiltrated by T or NK cells that secrete granzyme B [46]. Similar results were reported in another study, which may be associated with induced increase of tumour-specific CD8^+^ T cells [47,48]. Further, this might be related to increased CD4^+^ T cells [49] and pro-inflammatory TAMs [50], but a decrease of myeloid-derived suppressor cells (MDSCs), T regulatory cells (Tregs), and anti-inflammatory TAMs [9,48,51,52]. In the poorly immunogenic 4T1 breast cancer model, nsPEFs were shown to induce immune memory response by increasing CD4^+^ effector memory (CD44^+^CD62L^−^) and CD8^+^ central memory (CD44^+^CD62L^+^) T lymphocytes that were shown to be highly cytotoxic because of produced IFNγ [9]. Additionally, it was shown that local treatment with nsPEF significantly reduced 4T1 metastases compared to tumour-bearing control mice [9,52]. These studies have shown that the immune system is crucial for the progression of tumorigenesis. Evidence regarding nsPEF usage for tumour ablation and its effects on the immune system are increasing, although the exact mechanisms are intricate.

This work is based on our previous study [8,35], where we have shown the promising effects of nsPEF, however here we focus on the immunomodulatory effects of nsPEF-based procedures.

## 2. Materials and Methods

### 2.1. Generation of Luciferase Expressing LLC1 Carcinoma Cells

C57BL/6J mouse LLC1 carcinoma cells (the National Cancer Institute, Vilnius, Lithuania) were maintained in RPMI 1640 medium supplemented with 10% of foetal calf serum (FCS), 2 mM glutamine, 100 mg/mL streptomycin, and 100 U/mL penicillin (reagents were obtained from Thermo Fisher Scientific, Grand Island, NY, USA). The cells were cultured, maintained at 37 °C, 5% CO2. LLC1 cells were electrotransfected (4 × 100 µs × 1.2 kV/cm) with Bgl II-linearized Luciferase-pcDNA3 plasmid, a kind gift from William Kaelin (Addgene plasmid #18964, Harvard Medical School, Boston, MA, USA) [53]. G418 Sulphate (400 µg/mL) was used to select transfected LLC1 cells (Carl Roth GmbH, Karlsruhe, Germany). The cells that survived G418 selection were cloned into 96-well plates by limiting dilution. An in vitro bioluminescence assay was used to test if individually grown clones expressed luciferase. In this regard, half of the cells were transferred to white 96-well plates, adding D-Luciferin (Promega, Madison, WI, USA) to a final concentration of 150 µg/mL. Synergy 2 multi-modal microplate reader and Gen5 software (BioTek, Winooski, VT, USA) were used to evaluate the luminescence of LLC1 clones, measuring the total luminescence every 10 min for 4 h, at 37 °C. The best luminescent cell clones were selected by comparing the maximal luminescence (in RLU) of the clone’s overall kinetic reads. Luciferase-expressing cells were propagated and frozen in a medium containing 10% of DMSO and 90% of FCS, maintained in liquid nitrogen prior to use. The established luciferase-expressing cell line was named as LLC1-Luc [8].

### 2.2. Mice and Tumour Induction

C57BL/6J linear *Mus musculus* (further—mice) were bred and housed in the mouse facility of the State Research Institute Centre for Innovative Medicine (Vilnius, Lithuania). In this study, tumours were induced in 6–8-week-old mice by subcutaneously injecting 1 × 10^6^ of LLC1-Luc carcinoma cells resuspended in phosphate-buffered saline (PBS). When the tumours reached 100 mm^3^ (after 1–2 weeks), treatment was applied. The sizes of tumours were evaluated by volumetric measurements prior to and every 2–3 days after the treatment. Mice were kept till the end of the experiment (or when primary tumours had reached 3000 mm^3^). If so, mice were immediately sacrificed by cervical dislocation. Mice that fully recovered after the treatment were kept up to 72 days. Spleens and tumours were isolated and further used for multicolour flow cytometry. Blood was collected from the hearts of sacrificed mice, left to clot and centrifuged to obtain the serum that was further used for the determination of specific antitumour antibodies. The consent to perform animal experiments was obtained from the State Food and Veterinary Service (approval no. G2-145), carrying out the study strictly according to the Guide for the Care and Use of Laboratory Animals.

### 2.3. Electroporation

In the experiment, the square-wave pulse generator developed in VilniusTECH University (Vilnius, Lithuania) was used [53]. The pulses were delivered using adjustable parallel plate stainless steel electrodes (a 3–4 mm gap between electrodes was used for the electroporation of tumours). Electroporation was performed by compressing the tumour between the flat electrodes lubricated with EEG and ECG Transound gel (EF Medica Srl, Italy) to ensure good electrical contact. The voltage has been adjusted to ensure electric field generation of 3.5 kV/cm for nsECT and 1.3 kV/cm for ESOPE-based protocol (electric field was estimated as V/d, where V—measured voltage, d—gap between electrodes). The duration of the pulses was 200 ns, 700 ns (nsECT) and 100 µs (ESOPE). The pulses were delivered in bursts of 200 at 1 kHz or 1 MHz during nsECT procedure and in a burst of 8 with 1 Hz repetition frequency in the case of ESOPE protocol. The summary of electroporation protocols is shown in Table 1 [8]. The energy of the burst was estimated by multiplying the amplitudes of the current and voltage measurements, pulse duration and the number of pulses. It should be noted that only one instance in each group was evaluated and the effects of the electrode gap on the pulsed current were not taken into account. A total of 5 different protocols were employed.

### 2.4. Experimental Scheme

**Figure 1:**
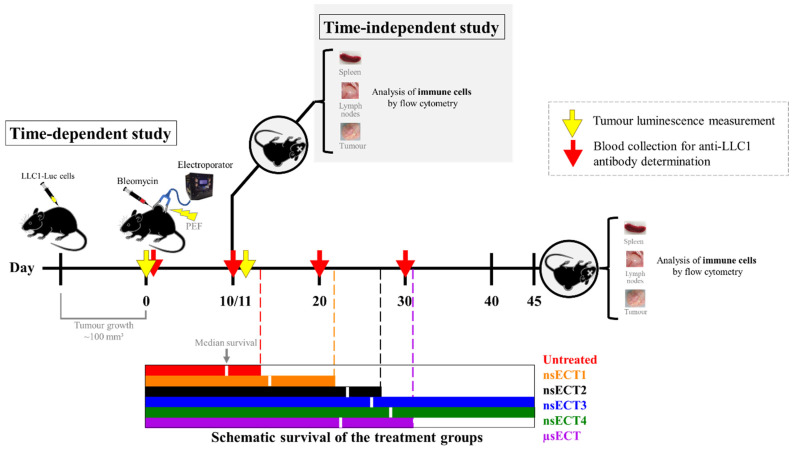
Schematic overview of the time-dependent and time independent studies.

In a time-dependent study, mice were divided into 6 groups: 1 control group of untreated tumour-bearing mice (no bleomycin or ECT treatment) and 5 treatment groups. Before the ECT treatment (day 0), at first, mice are put under anaesthesia of a 3% isoflurane and oxygen gas mixture. When mice are finally anaesthetized, concentration of isoflurane is lowered to 1.5%. Then, the backs of mice were shaved, depilated using 8% Na_2_S aqueous solution and then rinsed with water. Before the electroporation, a single injection of bleomycin solution (1500 IU in 50 μL of sterile PBS) was delivered into the tumours. After 5 min, mice were treated with PEF. The experimental design is shown in Figure 1. In a time-dependent study, tumour-bearing mice were kept until the tumor reached >3000 mm^3^ and then sacrificed.

Additionally, a time-independent study was performed to assess the changes in immune cell populations and antitumour antibodies, after nanosecond and microsecond ECT modalities. In this experiment, mice were divided into four groups—healthy mice (*n* = 6), untreated tumour-bearing mice (Untreated, *n* = 10), μsECT-treated (*n* = 11), nsECT4-treated (*n* = 11) mice (Table 1). For all ECT treatment modalities, bleomycin was used (1500 IU in 50 μL of sterile PBS). All mice were sacrificed 11 days after the treatment. The main goal of this study was to make a more in-depth analysis of changes in the immune system and antitumour IgG antibodies 11 days after the treatment regarding the differences observed in the time-independent study with the most successful protocols. Moreover, this study set up was chosen to catch early changes of forming the immune response in lymphoid organs. It should be noted that there was no experimental group focused on the chemotherapy with BLM alone since the main goal was to characterize the strongest immune response of the synergistic treatment (i.e., electrochemotherapy).

### 2.5. Evaluation of Tumour Sizes

Luminescence and volumetric measurements were used to assess the growth dynamics of tumours. After PEF treatment, the volume of tumours (or forming scabs) was determined by measuring centered tumour length and width with a digital caliper every 2–3 days after the treatment. The volumes of tumours (mm^3^) were calculated by the formula: V = (Length × Width^2^ × π)/6, where π = 3.1416. When the tumour volume reached about 3000 mm^3^, the mice were sacrificed by cervical dislocation.

The luminescence of tumours was assessed by imaging tumours with IVIS Spectrum equipment and Living Image Software (Caliper/Perkin Elmer, Akron, OH, USA). Prior to the treatment, mice were intraperitoneally injected with 150 µL (30 mg/mL in PBS) of D-luciferin solution (Promega, Madison, WI, USA). After 10–15 min, mice were visualised under anaesthesia with 3% isoflurane and oxygen gas mixture that was later lowered to 1.5% (Vetpharma Animal Health, S.L., Barcelona, Spain). Then, tumours were imaged before the treatment, right after the bleomycin and PEF treatment, as well as 11 days after the treatment. The bioluminescence of tumours was proportional to the number of live LLC1-Luc cells. Luminescence was expressed as the photons/sec/region of interest (ROI) by subtracting the background luminescence of the same size region.

### 2.6. Flow Cytometry

Tumours, spleens, and lymph nodes were mashed through a cell strainer into a 3.5 cm Petri dish with RPMI medium. Cells were centrifuged at 300× *g* for 5 min at room temperature (RT). Tumour cells were resuspended in a small amount of buffer for flow cytometry (2% foetal bovine serum (FBS) and 0.1% NaN_3_ in phosphate buffer saline (PBS)). Before centrifugation, splenocytes were resuspended in 5 mL of 0.16 M NH_4_Cl to lyse erythrocytes and incubated for 5 min. Then, 10mL of RPMI was added and centrifuged cells were resuspended in the FACS buffer. Cell surface staining was performed by incubation of 0.5 × 10^6^ of cells in 20 μL of FACS buffer with 20 μL of Master mix. Prepared samples were incubated on ice, away from light, for 30 min. Cell populations were identified by using different sets of fluorochrome-labelled antibodies and fluorescent dyes (staining strategies—further referred to as Staining 1, 2A/B, 3A/B/C/D, and 4A/B/C). The measurement and analysis of immune organs were performed with BD FACS Aria III instrument (BD Biosciences, San Jose, CA, USA). The staining, used antibodies, gating and analysis strategies are presented in the Supplementary Data, Figures S2–S6, and Table S1.

### 2.7. Determination of Specific Antitumour Antibodies

Mice blood sera were collected every 10 days after the treatment for the determination of antibodies against the surface and intracellular antigens of LLC1 cells. LLC1 cells were fixed with RT 2% paraformaldehyde in PBS buffer for 10 min at 37 °C, afterwards centrifuged at 300× *g* for 5 min at 4 °C and permeabilized with 0.2% ice-cold Triton X-100 in PBS for 9 min. After permeabilization, cells were immediately resuspended in FACS buffer and centrifuged at 300× *g* for 5 min at 4 °C. The LLC1 cells were filtered through a 70 µm strainer and diluted in Fc Block to 0.3 × 10^6^ cells per sample. Cells were incubated for 1 h in mice sera, which was stepwise diluted in PBS. Afterwards, cells were washed with PBS buffer, centrifuged at 300× *g* for 5 min at 4 °C and incubated with a goat anti-mouse IgG AF488 (eBioscience, Invitrogen, Thermo Fisher Scientific, Waltham, MA, USA) for 30 min on ice, away from light. Cells incubated with only goat anti-mouse IgG AF488 antibodies served as a negative control. The measurement of cells was performed using the Amnis FlowSight cytometer (Amnis Luminex/MilliporeSigma, Burlington, MA, USA), and data analysis software FlowJo (BD, USA). 

### 2.8. Statistical Analysis

All experimental data were analysed using GraphPad Prism 8 software (GraphPad Software Inc., La Jolla, San Jose, CA, USA). Non-parametric (two-tailed) Mann-Whitney-Wilcoxon test was used to determine if tumour volumes and cytometry results of different organs (spleens, tumours) significantly differed among groups of ECT-treated and untreated mice. Non-parametric (two-tailed) Wilcoxon matched-pairs test was used to compare the tumour volume values within the group at different times after treatment. Log-rank (Cox-Mantel) and Gehan-Breslow-Wilcoxon tests were used to analyse mice survival data (Kaplan-Meier survival analysis), comparing groups of ECT-treated mice among each other and to the group of untreated mice.

ECT-treated tumour-bearing mice groups compared to untreated tumour-bearing mice group: + *p* = 0.05–0.1 (tendency); * *p* < 0.05; ** *p* < 0.005; *** *p* < 0.0005 (marked in red) (statistically significant differences). nsECT-treated mice groups compared to μsECT-treated mice group: **🇴** *p* = 0.05–0.1 (tendency); ● *p* < 0.05; ●● *p* < 0.005 (statistically significant differences).

The summary of all the statistically significant changes (*p* < 0.05) in immune cell populations or immune cell surface markers, compared to untreated tumour-bearing mice, is presented in Tables S3 and S4 (Supplementary Material).

## 3. Results

### 3.1. Bleomycin ECT Decreased Tumour Growth and Increased Mice Survival

**Figure 2:**
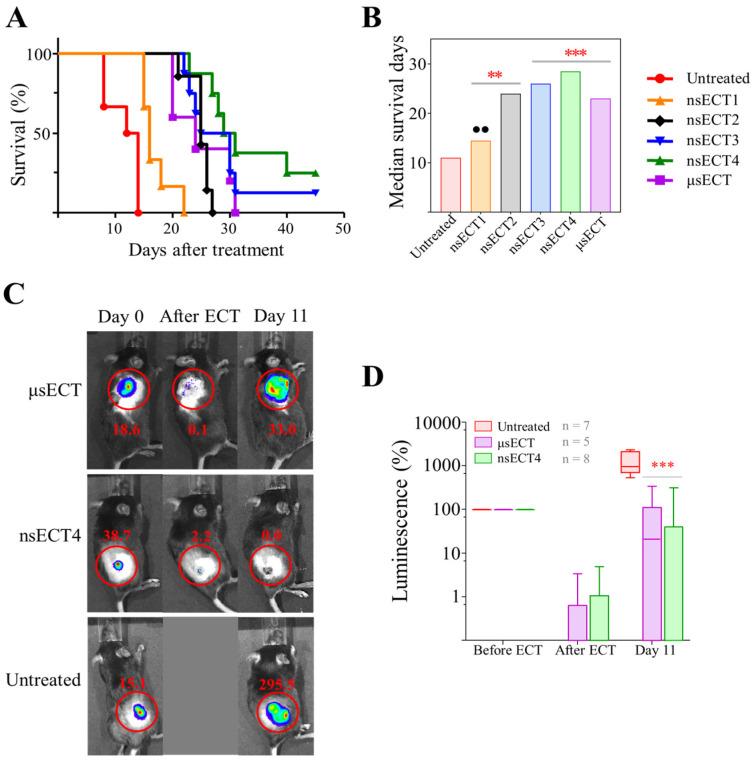
(**A**) Mice survival curves. (**B**) Median survival days. Mantel–Cox test was used for the statistical evaluation of mice survival. (**C**) Bioluminescence imaging of untreated, microsecond and nanosecond ECT treated tumours in tumour-bearing mice. (**D**) Tumour luminescence % before (Day 0), after ECT and 11 days after the treatment. The imaging in (**C**,**D**) was done with a IVIS Spectrum device and Living Image software. The Mann-Whitney test was used for the comparison of tumour luminescence data. Statistically significant (** *p* < 0.005; *** *p* < 0.0005) differences compared to the untreated group. Significant differences (●● *p* < 0.005) compared to the μsECT group.

In a time-dependent study, we assessed the effect of microsecond and nanosecond ECT on C57BL/6J mice survival. The results are summarized in Figure 2.

We have also used the luminescent LLC1-Luc cell line for the induction, visualization and tracking of tumours in vivo. Mice tumours were visualized before and after ECT (Day 0) and at Day 11 (Figure 2C,D). A statistically significant decrease in tumour luminescence was observed in ETC-treated compared to untreated tumour-bearing mice. 

At the end of the experiment, untreated and treated groups of C57BL/6J mice were further compared by survival (Figure 2A,B). Compared to untreated tumour-bearing mice, the survival of nsECT2, nsECT3, nsECT4 and μsECT-treated mice was significantly longer. The median survival days of untreated tumour-bearing mice was 11 days, whereas the median survival of ECT-treated mice was approximately 1.3–2.5 times longer (nsECT1—14.5 days; nsECT2—24 days, nsECT1—26 days, nsECT4—28.5 days, and μsECT-treated—23 days). Only in nsECT3 and nsECT4 treatment groups, 12.5% and 25% of mice, respectively, had no tumours at the end of the experiment.

### 3.2. Electrochemotherapy with Bleomycin Modulates Cellular Immunity

Firstly, mice survival rates were assessed between four different nsECT treatment groups and compared to the μsECT treatment or untreated tumour-bearing mice group. During the first experiment, preliminary changes of splenic and tumour-associated immune cell populations and antitumour antibodies were assessed (time-dependent study). Then, according to the obtained results, the best treatment parameter (nsECT4; 3.5 kV/cm × 700 ns × 200 pulses, 1 MHz) was selected for further immunological time-independent study and compared to the standard ESOPE protocol.

**Figure 3:**
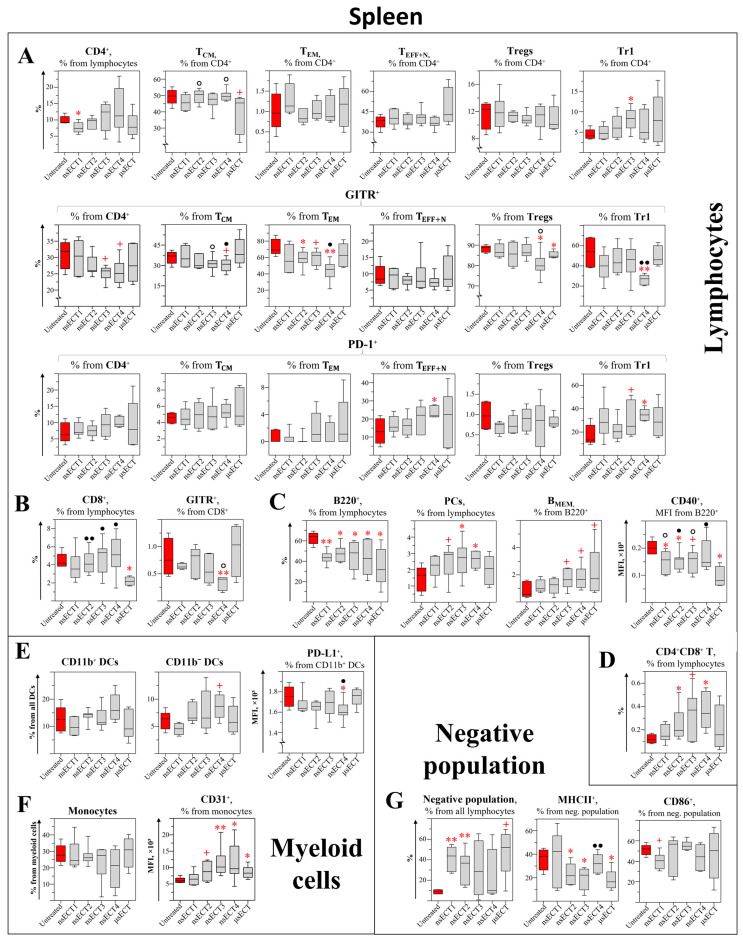
Time-dependent study. Lymphocyte (**A**–**D**), myeloid cells (**E**,**F**) and “Negative” (Lin^−^) population (**G**) in mice spleens. Cytometry was performed with BD FACSAria III cytometer. Statistically significant (* *p* < 0.05; ** *p* < 0.005) differences and tendencies (+ *p* = 0.05–0.1) compared to the untreated group. Significant differences (● *p* < 0.05; ●● *p* < 0.005) and tendencies (🇴 *p* = 0.05–0.1) compared to the μsECT group.

At the end of the experiment, both untreated tumour-bearing and ECT-treated mice with regrowing tumours had enlarged spleens (Figure S1). Moreover, untreated tumour-bearing mice had a higher percentage of erythrocytes compared to healthy or ECT-treated ones. However, healthy and ECT-treated mice with successfully eliminated tumours had normal-sized spleens. To assess what influences spleen volumes, we aimed to make an in-depth analysis of changes in splenic immune populations and their surface markers (Figure 3).

Firstly, changes in splenic T lymphocyte subpopulations were analysed. In a time-independent study, after ECT treatment, we observed a normalised percentage of CD3^+^ lymphocytes that matches with healthy mice.

**Figure 4:**
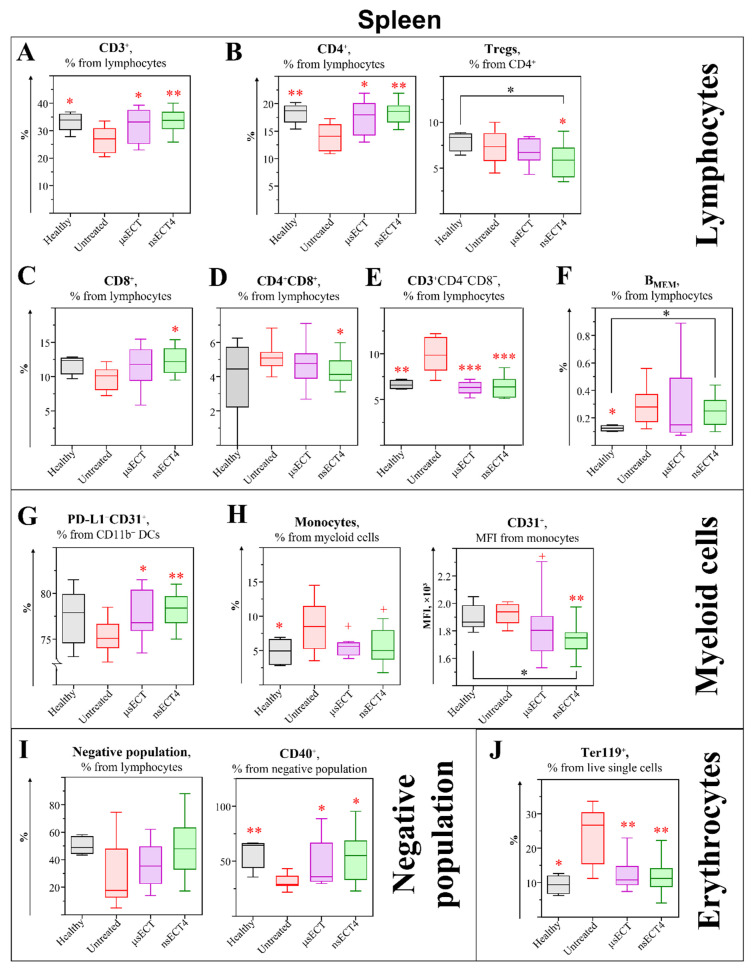
Time-independent study. Lymphocytes (**A**–**F**), myeloid cells (**G**,**H**), “Negative” (Lin^−^) population (**I**) and erythrocytes (**J**) in mice spleens. Cytometry was performed with BD FACSAria III cytometer. Statistically significant (* *p* < 0.05; ** *p* < 0.005; *** *p* < 0.0005) differences and tendencies (+ *p* = 0.05–0.1) compared to the untreated group.

Analysis of CD4^+^ subsets. Changes of splenic CD4^+^ lymphocyte subpopulations were analysed in a time-dependent study (Figure 3A–D and Figure 4A,B). A significant decrease in the percentage of all CD4^+^ T cells was observed in spleens of nsECT1-treated, compared to untreated, mice (Figure 3A). However, in a time-independent study, 11 days after ECT treatment, the percentage of CD4^+^ T cells had equalized with the percentage observed in healthy mice (Figure 4B). Furthermore, in time-dependent study a tendentious increase of CD4^+^ central memory T (Figure 3A; T_CM_; CD3^+^CD4^+^FR4^+^CD25^−^) cells was observed in nsECT2 and nsECT4-treated, compared to the standard microsecond treatment group, and tendentiously decreased in μsECT-treated mice, compared to untreated ones. GITR expression on T_CM_ cells was significantly decreased in nsECT4, compared to μsECT-treated mice. However, GITR expression on effector memory CD4^+^ T (Figure 3A; T_EM_; CD3^+^CD4^+^FR4^−^CD25^+^) cells was decreased in nsECT2, nsECT3 and nsECT4-treated, compared to untreated mice. However, only nsECT4-treated mice had lower expression levels of GITR on T_EM_ population, compared to standard microsecond ECT. Significantly decreased expression levels of GITR were observed on CD4+ regulatory T cells (Figure 3A; Tregs; CD3^+^CD4^+^FR4^+^CD25^+^) in nsECT4 and μsECT-treated compared to untreated mice. Furthermore, tendentiously lower expression levels of GITR were observed in nsECT4-treated compared to μsECT-treated mice. Moreover, a significant decrease in CD4^+^ Tregs was observed already 11 days after the treatment in nsECT4-treated mice compared to healthy and untreated tumour-bearing mice (Figure 4B). In a time-dependent study, an increase of CD4^+^ Type 1 regulatory T (Tr1; CD3^+^CD4^+^CD49b^+^) cells was observed in nsECT3-treated mice compared to untreated ones. A significant decrease in GITR expression levels was observed on Tr1 cells in nsECT4-treated mice, compared to μsECT-treated and untreated mice. Moreover, a significant increase in PD-1 expression levels was observed on Tr1 and effector naïve (T_EFF+N_; CD3^+^CD4^+^FR4^−^CD25^−^) T cells, in nsECT4-treated compared to untreated mice (Figure 3A).

Analysis of CD8^+^ subsets. A significant increase of cytotoxic T cells (Tc; CD3^+^CD8^+^) was observed in a time-independent study in nsECT4-treated mice (Figure 4C). However, in a time-dependent study, we observed no significant differences in the percentage of CD8^+^ T cells in nsECT4-treated compared to untreated or healthy mice. However, a significant decrease of Tc cells was observed after μsECT treatment, in comparison to other treatment modalities where Tc cells did not decrease (Figure 3B). The percentage of Tc cells was not decreased after nsECT2, nsECT3 and nsECT4 treatment, compared to the decrease that was visible in the spleens of μsECT-treated mice. The expression levels of Glucocorticoid-Induced TNFR-Related (GITR) Protein were significantly decreased on Tc cells in nsECT4-treated mice, compared to untreated mice (Figure 3B). Supplementary data on splenic CD8^+^ T cells is provided in supplementary material (Figures S7 and S8).

Analysis of double-positive (CD4^+^CD8^+^) and double-negative (CD4^−^CD8^−^) T cell subsets. A significant increase of double positive T cells (DP T; CD3^+^CD4^+^CD8^+^) were observed in only two modalities of nanosecond treatment groups (nsECT2 and nsECT4), compared to untreated mice group (Figure 3D). On the contrary, a significant decrease of DP T cells was observed in nsECT4-treated compared to untreated tumour-bearing mice, 11 days after the treatment (Figure 4D). In a time-independent study, a significant decrease of double-negative T cells (DN T; CD3^+^CD4^−^CD8^−^) was observed in all ECT treated and healthy groups when compared to untreated tumour-bearing mice (Figure 4E).

Analysis of B cell subsets. The percentage of B cells (CD3^−^CD19^+^B220^+^) was significantly decreased in all ECT-treated mice (Figure 3C). CD40 expression levels were significantly lower in nsECT1, nsECT2 and μsECT-treated compared to untreated mice. Whereas nsECT2 and nsECT4-treated mice had significantly higher expression levels of CD40 on B cells, compared to μsECT-treated mice. However, plasma cells (PCs; CD3^−^CD138^+^) were significantly increased in nsECT3 and nsECT4-treated mice compared to untreated ones. Memory B cells (B_MEM_; B220^+^CD19^+^CD27^+^) are tendentiously increased in nsECT3, nsECT4 and μsECT-treated mice, compared to untreated mice. Similarly, in a time-independent study, 11 days after nsECT4 treatment, a significant increase of B_MEM_ cells was observed compared to healthy mice (Figure 4F).

Analysis of myeloid cells. No significant changes were observed in the percentage of CD11b^+^ DCs (CD3^−^B220^−^CD11c^+^CD11b^+^) and CD11b^−^ DCs (CD3^−^B220^−^CD11c^+^CD11b^−^) in spleens, comparing untreated and ECT-treated mice. After nsECT4 treatment, PD-L1 expression levels are significantly decreased on CD11b^+^ DCs (Figure 3E). In time-independent study, increased expression of CD31 and decreased expression of PD-L1 was observed on CD11b^−^ DCs after ECT treatment compared to healthy or untreated tumour-bearing mice. In a time-independent study, a decrease of splenic monocytes (CD11b^+^Gr1^−^) was observed in ECT treated and healthy mice, compared to untreated tumour-bearing mice (Figure 4H). Moreover, increased expression of CD31 was observed on monocytes after ECT treatment in time-independent study (Figure 3F). On the contrary, 11 days after the treatment, a significant decrease of monocytes CD31 was observed in nsECT4-treated mice, compared to healthy and tumour-bearing mice (Figure 4G).

Analysis of the “Negative” (Lin^−^) population. We identified a lineage-negative immune cell population CD45^+^CD3^−^/4^−^/8^−^B220^−^CD19^−^Gr-1^−^CD11c^−^CD11b^−^CD49b^−^CD1d^−^CD88^−^FR4^−^CD62L^−^TCRγ/δ^−^Ter119^−^CD43^−^ (Lin^−^) in spleens, further in the text referred as “negative” (Lin^−^) population (Figure 3G). According to the SSC-A and FSC-A ratio, these cells were distributed within the lymphocyte gate. This Lin^−^ population had a weak (0–15%) expression of CD5, F4/80, CD44, GITR, CD49b, CD40 markers and a higher expression level of MHCII (5–45%), CD80 (10–50%), PD-L1 (20–77%), CD163 (20–78%), CD86 (30–85%), CD24 (42–98%) (Table S2). 

The percentage of splenic Lin^−^ population in time-dependent study was significantly increased after nsECT1 and nsECT2 treatment. However, this population had significantly lower expression levels of MHCII in nsECT2, nsECT3 and μsECT-treated mice, compared to untreated tumour-bearing mice. Thus, the expression levels of MHCII on the Lin^−^ population were not decreased after nsECT4 treatment, compared to standard μsECT treatment. Moreover, the expression levels of CD86 on Lin^−^ population were tendentiously decreased in nsECT1-treated mice, compared to untreated mice (Figure 3G). However, in time-independent study, the expression levels of CD40 on Lin^−^ population were significantly increased, compared to untreated tumour-bearing mice (Figure 4I).

After the ECT treatment, the percentage of erythrocytes has normalized and is comparable with healthy mice (Figure 4J).

**Figure 5:**
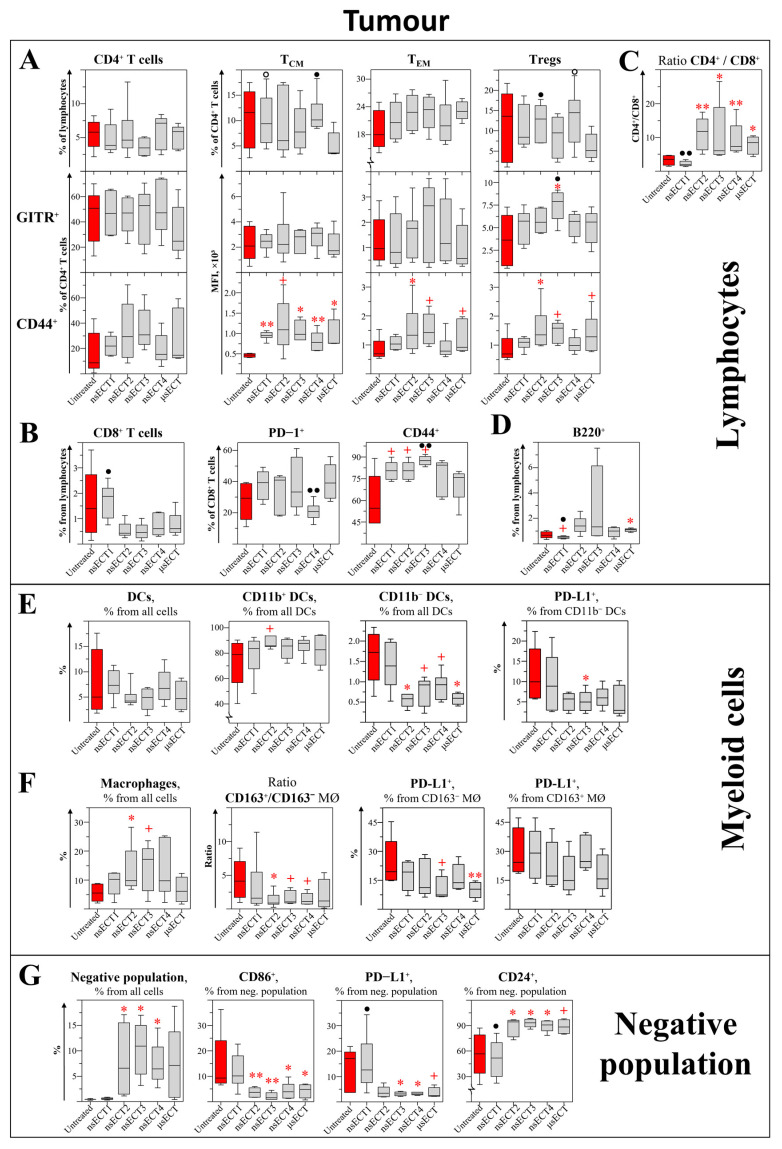
Time-dependent study. Lymphocyte (**A**–**D**), myeloid cells (**E**,**F**) and “Negative” (Lin^−^) population (**G**) in mice tumours. Cytometry was performed with BD FACSAria III cytometer. Statistically significant (* *p* < 0.05; ** *p* < 0.005) differences and tendencies (+ *p* = 0.05–0.1) compared to the untreated group. Significant differences (● *p* < 0.05; ●● *p* < 0.005) and tendencies (**🇴** *p* = 0.05–0.1) compared to the μsECT group.

**Figure 6:**
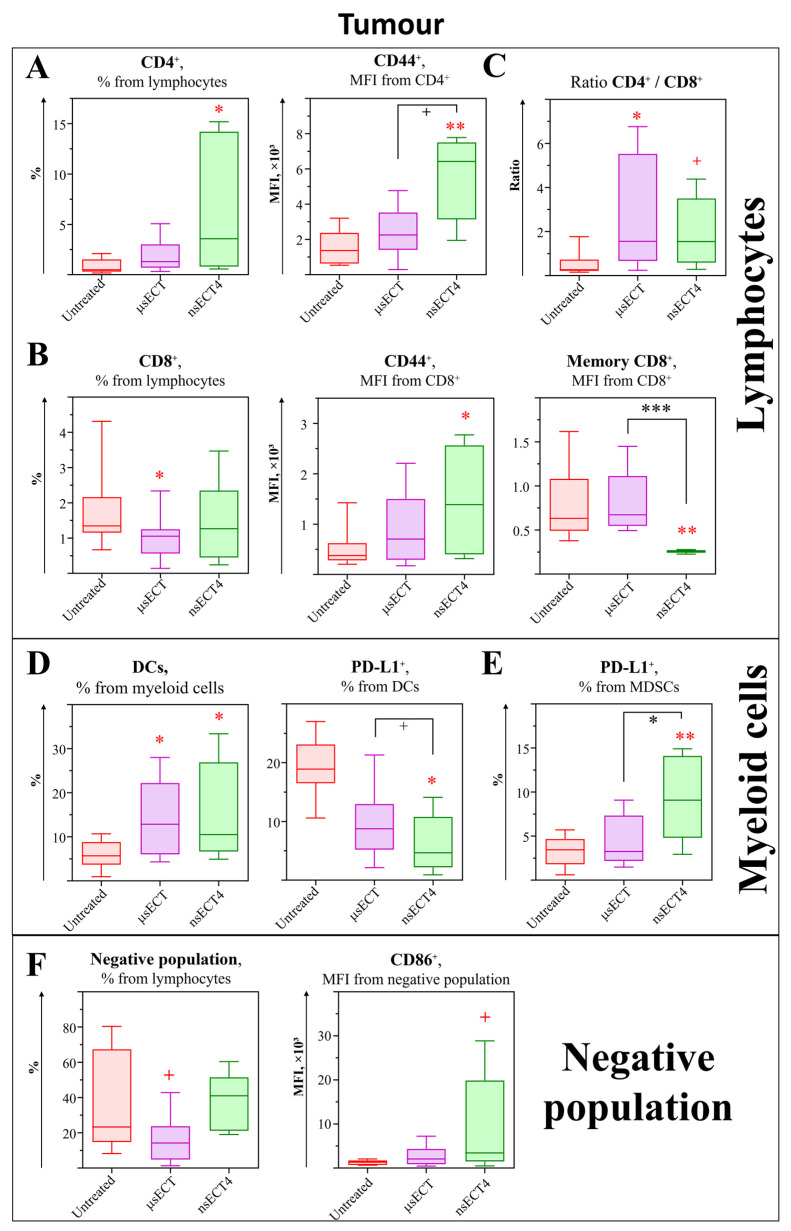
Time-independent study. Lymphocytes (**A**–**C**), myeloid cells (**D**,**E**) and “Negative” (Lin^−^) population (**F**) in mice tumours. Cytometry was performed with BD FACSAria III cytometer. Statistically significant (* *p* < 0.05; ** *p* < 0.005; *** *p* < 0.0005) differences and tendencies (+ *p* = 0.05–0.1) compared to the untreated group.

Analysis of CD4^+^ subsets. Further changes of immune cells in tumours were assessed (Figure 5 and Figure 6). In a time-dependent study, no significant differences were observed among tumour-associated CD4^+^ T cell subsets in ETC-treated mice compared to untreated tumour-bearing mice (Figure 5A). However, in the time-independent study, a significant increase of tumour CD4^+^ T cells and increased expression levels of CD44 marker on these cells were observed in nsECT4-treated compared to untreated tumour-bearing mice (Figure 6A). In the time-dependent study, a significant increase of CD4^+^ T_CM_ cells was observed in nsECT4-treated, compared to μsECT-treated mice. Subset-wise, a significant increase in the expression levels of CD44 was observed on CD4^+^ T_CM_ cells in all ECT-treated compared to untreated mice. The expression levels of GITR were significantly increased on CD4^+^ Tregs of nsECT3-treated mice compared to untreated ones. Moreover, expression levels of CD44 were increased on T_EM_ and Tregs, significantly in nsECT2-treated and tendentiously in μsECT and nsECT3-treated mice, compared to untreated ones (Figure 5A). Notably, a significantly increased CD4^+^ T to CD8^+^ T cell ratio was observed in all ECT-treated groups except nsECT1 (Figure 5C). Similarly, in the time-independent study, 11 days after ECT treatment, a significant increase of CD4^+^ T to CD8^+^ T cells (according to CD4^+^/CD8^+^ ratio) was observed compared to untreated mice (Figure 6C).

Analysis of CD8^+^ subsets. Results of CD8^+^ cytotoxic T cells analysis can be seen in Figure 5B. No significant changes were observed in the percentage of Tc cells in ECT-treated compared to untreated mice. On the contrary, a significant decrease of cytotoxic T cells was observed in μsECT-treated compared to untreated tumour-bearing mice, 11 days after the treatment (Figure 6B). Moreover, we observed an increased expression levels of activation marker (CD44) on cytotoxic tumour-associated T cells, in nsECT4-treated compared to untreated tumour-bearing mice (Figure 6B). In the time-dependent study, expression levels of PD-1 were diminished in CD8^+^ T cells in nsECT4-treated compared to μsECT-treated mice. Whereas nsECT2 and nsECT3-treated mice had a higher expression level of CD44 on cytotoxic T cells. However, in the time-independent study, a significant decrease of tumour-associated memory Tc cells (CD44^+^CD62L^+^CD8^+^) was observed, in nsECT4-treated compared to μsECT and untreated tumour-bearing mice (Figure 6B). Supplementary data on cytotoxic tumour-associated T cells is provided in supplementary material (Figures S7 and S8).

Analysis of B cell subsets. A significant increase in the percentage of B cells (CD3^−^B220^+^CD19^+^) was observed in μsECT-treated mice compared to untreated tumour-bearing mice (Figure 5D). B cells were decreased in the nsECT1-treated compared to μsECT-treated and untreated mice. 

Analysis of myeloid cells. Subsets of tumour-associated dendritic cells and macrophages as well as their surface marker expression levels were observed (Figure 5E). A significant decrease of CD11b^−^ DCs was observed in nsECT2 and μsECT-treated mice, compared to untreated tumour-bearing mice. Thus, in the time-independent study, a significant increase of DCs in tumours was observed after μsECT and nsECT4 treatment, compared to untreated mice. Also, decreased expression of PD-L1 was observed on tumour associated DCs after nsECT4 treatment compared to untreated tumour-bearing mice (Figure 6D). Moreover, in the time-independent study, significantly decreased expression levels of PD-L1 were observed on CD11b^−^ DCs in nsECT3-treated mice. The percentage of tumour-associated macrophages (TAM; CD3^−^B220^−^Gr1^−^CD11b^+^F4/80^+^) was significantly increased in nsECT2 and tendentiously increased in nsECT3-treated compared to untreated mice (Figure 6F). However, PD-L1 expression levels on pro-inflammatory (CD163^−^) TAMs were significantly decreased in μsECT-treated and tendentiously decreased in nsECT3-treated mice, compared to untreated ones. According to the ratio of anti-inflammatory (CD163^+^) to pro-inflammatory TAMs, a significant skew towards the pro-inflammatory phenotype was observed in the nsECT2-treated group and tendentiously decreased in nsECT3 and nsECT4-treated mice, compared to untreated tumour-bearing mice (Figure 5F). In the time-independent study, increased expression levels of suppressor PD-L1 marker on tumour-associated myeloid-derived suppressor cells (MDSCs; CD11b^+^Ly-6G^+^Ly-6C^−^) was observed only in nsECT4-treated compared to untreated and μsECT-treated mice (Figure 6E).

Analysis of the “Negative” (Lin^−^) population. The previously described Lin^−^ population was also assessed in tumours (Figure 5G). Tumour- associated Lin^−^ population was significantly increased in all nsECT-treated mice groups, except nsECT1, compared to the untreated group. This population had decreased expression levels of CD86 and increased expression levels of CD24 in ECT-treated compared to untreated mice. However, the expression levels of PD-L1 were only decreased in this population in the nsECT3 and nsECT4-treated mice groups, compared to the untreated tumour-bearing mice group. On the contrary, the percentage of tumour Lin^−^ population in the time-independent study was only significantly decreased after μsECT treatment (Figure 6F). However, this tumour-associated population had significantly higher expression levels of CD86 in nsECT4-treated mice, compared to untreated tumour-bearing mice, 11 days after the treatment.

**Figure 7:**
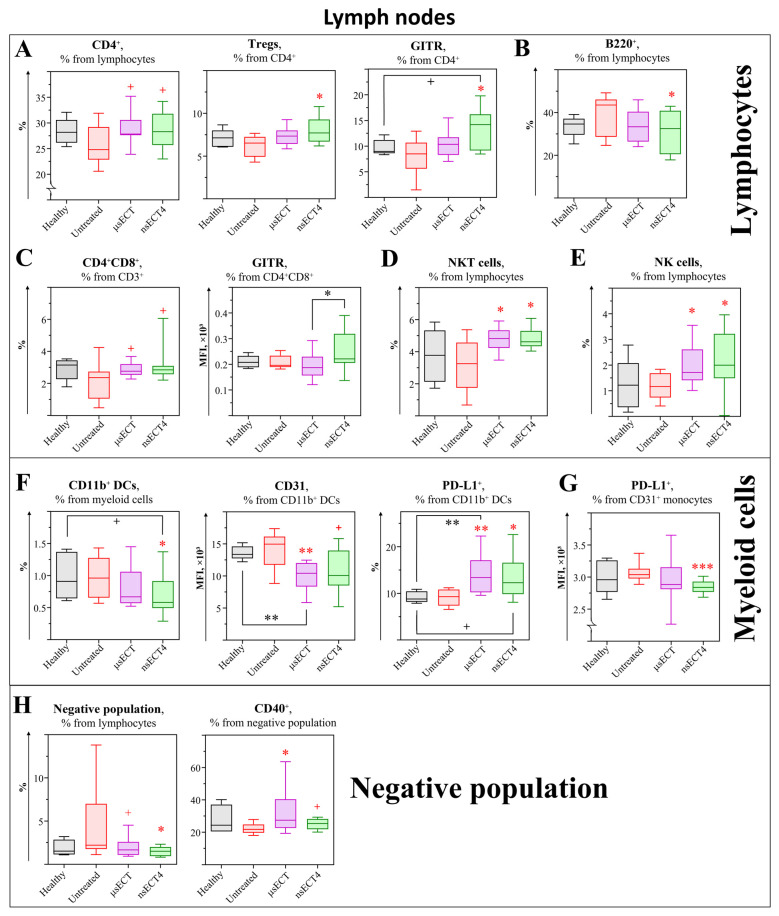
Time-independent study. Lymphocytes (**A**–**E**), myeloid cells (**F**,**G**) and “Negative” (Lin^−^) population (**H**) in murine lymph nodes. Cytometry was performed with BD FACSAria III cytometer. Statistically significant (* *p* < 0.05; ** *p* < 0.005; *** *p* < 0.0005) differences and tendencies (+ *p* = 0.05–0.1) compared to the untreated group.

Analysis of CD4^+^ subsets. In the time-independent study, the changes of immune cells were assessed in lymph nodes (Figure 7). A tendentious increase of helper T cells was observed in ETC-treated mice compared to untreated tumour-bearing mice. Additionally, an increase in the expression levels of the GITR marker was observed on CD4^+^ T cells in nsECT4-treated compared to untreated and healthy mice. Subset-wise, a significant increase of CD4+ Tregs was observed in nsECT4-treated compared to untreated mice (Figure 7A). Notably, a tendentious increase of double-positive T cells (CD4^+^ CD8^+^) was observed in all ECT-treated groups. Moreover, a significant increase in the expression levels of the GITR marker was observed on DP T cells in nsECT4-treated compared to μsECT-treated mice (Figure 7C). Supplementary data on cytotoxic T cells is provided in supplementary material (Figure S8).

Analysis of B cell subsets. In the time-independent study, a decrease in B cells (CD19^+^B220^+^) was observed in nsECT4-treated compared to untreated mice (Figure 7B). However, the percentage of B cells did not change after ECT treatment compared to the healthy mice group.

Analysis of NK and NKT cell subsets. An increase of NKT (Figure 7D) and NK (Figure 7E) cells was observed in ECT treated compared to healthy and untreated tumour bearing mice.

Analysis of myeloid cells. In the time-dependent study, a significant decrease of CD11b^+^ DCs was observed in nsECT4-treated compared to untreated mice (Figure 7F). However, the decrease of CD11b^+^ DCs in nsECT4-treated mice was only tendentious when compared to healthy mice. Moreover, a decrease in the expression levels of CD31 and increase in the expression levels of PD-L1 were observed in ECT-treated compared to untreated and healthy mice. However, compared to healthy mice, decreased expression of CD31 and increased expression of PD-L1 were significant only in μsECT-treated mice. PD-L1 was also assessed in monocytes (Figure 7G). A significant decrease in expression levels of PD-L1 was observed in nsECT4-treated compared to untreated mice.

Analysis of the Lin^−^ population. Lin^−^ population was decreased in the lymph nodes of ECT-treated compared to healthy and untreated tumour-bearing mice. However, compared to untreated tumour-bearing mice, an increase in the expression levels of CD40 was observed on this Lin^−^ population after ECT treatment (Figure 7H). 

### 3.3. Bleomycin nsECT STIMULATES the formation of Humoral Antitumour Immune Response

**Figure 8:**
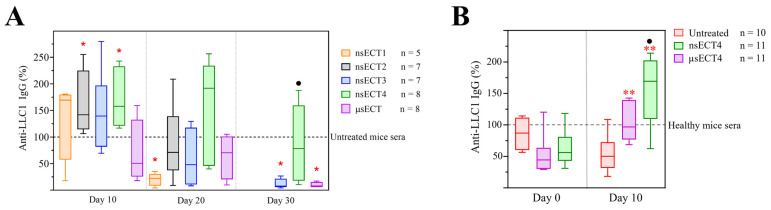
The relative percent of anti-LLC1 IgG antibodies in time-dependent (**A**) and time-independent (**B**) study. Data was normalized according to (*n* = 7) untreated tumour-bearing (**A**) and (*n* = 6) healthy mice (**B**). Statistically significant (* *p* < 0.05; ** *p* < 0.005) differences compared to untreated group; significant differences (● *p* < 0.05) compared to the μsECT group. Flow cytometry was performed with Amnis FlowSight.

According to the previously observed increase of plasma and memory B cells, the relative percent of anti-LLC1 IgG antibodies were further assessed (Figure 8). In the time-dependent study, nsECT2 and nsECT4 resulted in a significant increase of anti-LLC1 IgG antibodies 10 days after the treatment, compared to untreated tumour-bearing mice group (Figure 8A). Similar results of the relative percent of anti-LLC1 IgG antibodies were observed in a time-independent study, 11 days after ECT treatment (Figure 8B). Notably, after the nsECT4 treatment, the levels of antitumour IgG antibodies remained similarly high throughout the experiment. Thirty days after the experiment, the percentage of anti-LLC1 IgG was significantly higher in the sera of nsECT4-treated compared to untreated tumour-bearing mice. A decrease of antitumour IgG antibody levels was observed 20 days and 30 days after ECT, in comparison to 10 days.

## 4. Discussion

Previously, we have successfully shown that doxorubicin could be used in nano-electrochemotherapy to eliminate SP2/0 tumours in BALB/c mice [19]. Additionally, our group has applied calcium electroporation for the effective elimination of myeloma tumours where immune system changes were assessed [35]. This research focuses on in vivo biomedical applications of high-frequency nanosecond electroporation in the bleomycin electrochemotherapy context. 

In this study, tumour-bearing mice were treated with microsecond electrochemotherapy (μsECT) and different modalities of nanosecond electrochemotherapy (nsECT: 3.5 kV/cm × 200 pulses) and compared to untreated mice. Tumour luminescence and survival rates were assessed. We observed that nsECT-treated mice reached similar survival as μsECT-treated mice. Standard microsecond electrochemotherapy procedures are shown to eliminate tumours, or reduce their growth, in various studies [54,55,56], which is agreement with our data. However, currently not much research has been done on nanosecond electrochemotherapy, while recent works have shown that high-frequency electroporation can be as efficient as microsecond electrochemotherapy [10,57,58]. 

We observed that mice treated with nsECT1 (200 ns, 1 kHz) have shown only one third of the survival rate of mice, which were treated with standard ESOPE protocol based on μsECT. Compared to untreated mice or treated with nsECT1, mice treated with longer pulses nsECT3 (700 ns, 1 kHz) or high-frequency nsECT4 (700 ns, 1 MHz) have shown significantly longer survival. Moreover, after nsECT3 and nsECT4 treatment, 12.5% and 25% of mice, respectively, had successfully recovered and lived up to the end of the experiment. It can be noted that longer treatment duration (700 ns) was superior to 200 ns procedures (nsECT1, nsECT2), which is in agreement with established knowledge [14,59].

We have determined that changes in splenic and tumour-associated immune cell subpopulations occur in ECT-treated and untreated mice. We assessed that, after successful ECT treatment, mice had normal-sized spleens, similarly to healthy mice. At the end of the experiment, untreated tumour-bearing mice and ECT-treated mice with re-growing tumours had enlarged spleens. Mice that successfully recovered had normal-sized spleens compared with healthy mice. In time-independent study, we have shown that after the ECT treatment, the percentage of erythrocytes has normalized and is comparable with healthy mice (Figure 4J). Similar observations were described in literature noting that splenomegaly occurs because of metastasis, or splenic immune cell activation induced by blood-circulating tumour environment-related antigens or growth factors [60,61,62]. In our study, it can be seen that ECT treatment slowed down tumour growth and delayed the onset of splenomegaly. 

An increased expression of GITR was only observed on Tregs in tumours (nsECT3). However, the expression levels of CD44 were increased on tumour-associated T_EM_ and Tregs in nsECT2-treated mice. Some studies [63,64,65,66] have shown that GITR upregulation is typical for at least once activated and CD44—for reactivated effector T cells. Whereas PD-1 upregulation usually suppresses T cells. Also, GITR triggering and blocking of PD-1 receptor with antagonistic antibodies can result in activation of T cells and promotes antitumour immunity. Furthermore, it is suggested [67,68] that PD-1 and GITR expression could inversely correlate. In this study, decreased splenic CD4^+^ T cells in nsECT1-treated mice might have been related to slightly poorer adaptive antitumour immunity that can also be seen in the survival data. An increase in splenic Tr1 subset in nsECT3-treated mice might not be directly related to the long survival observed in this group. However, increased splenic regulatory cells might be related to the overstimulation of splenic immune cells by growth hormones coming from the tumour site and observed splenomegaly. Based on the findings of this study, it could be suggested that downregulated GITR and upregulated PD-1 expression on Tr1 cells would decrease the activation of these cells in nsECT4-treated mice. Moreover, decreased expression levels of GITR on splenic Tregs in ECT-treated mice could less suppress the activation of various immune cells. Less active splenic Tregs and Tr1 cells could less suppress innate and adaptive effectors of antitumour immunity, especially in the initial stage of regrowing tumour formation. Thus, more active effector cells could more efficiently repress regrowing tumours, as it was observed in nsECT3 and nsECT4 treatment groups. 

In both, time-dependent and time-independent studies, splenic CD8^+^ T (Tc) cells have increased in nsECT4-treated compared to untreated or μsECT-treated mice. A decrease of tumour-resident CD8^+^ T lymphocytes was observed in μsECT-treated compared to untreated mice in the time-independent study. Moreover, the expression of CD44 was upregulated on splenic CD8^+^ T lymphocytes, 11 days after the treatment. Also, a significant decrease of tumour-associated memory cytotoxic T cells (CD8^+^CD44^+^CD62L^+^) was observed after nsECT4 treatment. In time-dependent study, downregulated expression of GITR was observed on splenic Tc cells in nsECT4-treated mice. Whereas nsECT2 and nsECT3-treated mice had higher expression levels of CD44 and GITR on tumour-associated cytotoxic T cells. It was shown [65,66] that GITR upregulation is related to the activation of cytotoxic T cells. In our study, downregulation of GITR on splenic T cells might not be straightforward to understand. However, taking into consideration significantly prolonged survival of nsECT4-treated mice we speculate that it could be related to diminished memory cytotoxic T cells, increased GITR and CD44 activation on effector T cells and decreased activation of regulatory T cells. Moreover, the expression levels of PD-1 were diminished on Tc cells in nsECT4-treated compared to μsECT-treated mice, noting that these cells could be more active in the tumour microenvironment.

Additionally, in a time-independent study, NK (CD3^−^CD49b^+^) and NKT (CD3^+^CD49b^+^) cells were significantly increased in lymph nodes after ECT treatment compared to untreated tumour-bearing mice. According to the literature, NK and NKT cells play a role in controlling the lymphatic spread of cancer by killing metastatic cancer cells in tumour-draining lymph nodes [69,70,71,72,73].

In a time-dependent study, after nsECT4 treatment, an increase of splenic double-positive CD4^+^CD8^+^ T cells was observed. Oppositely, the percentage of DP T cells was decreased in time-independent study, matching the percentage observed in healthy mice. The expression levels of GITR were increased on CD4^+^CD8^+^ T cells in lymph nodes after nsECT4 treatment. Some researchers [74,75] have shown that DP T cells, due to frequent activation and increased transcriptional background, co-express CD4 and CD8 when they are exhausted. Such exhausted cells can have various functions. It was also shown [76] that tumour infiltrating CD4^+^CD8^+^ T can have a memory phenotype and can promote antitumour immunity. However, another group of researchers [77] has shown that CD4^+^CD8^+^ T cells can also have regulatory properties. In this study, considering the prolonged survival rates of nsECT2 and nsECT4-treated mice, an increase of CD4^+^CD8^+^ T cells might suggest that this subpopulation could positively modulate antitumour immunity. Moreover, in time-independent study, ECT-treated mice had decreased the percentage of splenic double-negative T cells (CD3^+^CD4^−^CD8^−^), which was nearly the same as in healthy mice. In the literature CD3^+^CD4^−^CD8^−^ subset is still poorly described. However, this CD3^+^ double-negative population is associated with homeostatic role in autoinflammatory conditions by suppressing excessive immune response against self-antigens. However, after ECT treatment, this DN T cell population could act as a cytotoxic, regulatory or helper T subset in tumorigenesis depending on the tumour microenvironment [78,79,80,81].

In the time-dependent study, we observed that B cells were decreased in spleens, but increased in tumours after ECT treatment. Furthermore, the expression levels of CD40 on splenic B cells were significantly increased in nsECT2 and nsECT4 compared to μsECT-treated mice. A few researchers [82,83] have shown that strong CD40 activation promotes B cell differentiation into memory B or plasma cells. However, some studies [84,85] have shown that the differentiation of tumour-associated B cells into regulatory or effector subsets depends on tumour microenvironment. Based on our data, we suggest that after ECT treatment an increased percentage of splenic plasma and memory B cells and upregulated expression of their activation markers might positively add up to forming a humoral immune response. Furthermore, we have shown that 10 days after ECT treatment, higher levels of anti-LLC1 IgG antibodies were formed in comparison to the μsECT treatment. According to some observations [86,87], we suggest that electroporation treatment releases tumour cell antigens that can be picked up by antigen presenting cells in the tumour microenvironment, where the antigen presentation could further launch the activation of other effector lymphocytes. We could see that nsPEF allow better modulation of the immune response, however, the levels of antitumour IgG antibodies are decreasing after some time, when comparison of the results from the day 10 to 20 days and 30 days after the ECT treatment is performed. As explained in a few studies [88,89], this might be related to the overall suppressive effect of chemotherapy on the immune system. Or short-lived plasma cells could have died off by the time long-lived plasma cells have formed that are lower in numbers [90,91]. 

Increased all tumour-associated dendritic cells and a decreased subset of CD11b^+^ DCs in tumour-draining lymph nodes were observed in nsECT4-treated mice compared to untreated tumour-bearing mice. Expression levels of PD-L1 were decreased on tumour-associated DCs and increased on a subset of CD11b^+^ DCs in the lymph nodes after ECT treatment. Moreover, a significant decrease in the expression levels of PD-L1 was observed on splenic CD11b^+^ DCs subset in nsECT4-treated mice, compared to μsECT-treated and untreated tumour-bearing mice. Additionally, an increased expression level of CD31 was observed on CD11b^−^ DCs in ECT-treated compared to untreated mice. CD31 on Dendritic cells work as an inhibitory molecule. Some researchers have shown that genetically modified dendritic cells lacking CD31 had increased potential to activate T lymphocytes [92]. Some studies [93,94,95] have shown that CD11b^−^, as well as CD11b^+^ DCs, migrate to tumour-draining lymph nodes, activate naïve T cells, and promote their expansion, leading to antitumour immunity. According to the literature, low migration of CD11b^−^ DCs and localization in tumours was related to expanded regulatory T cells, promoting tumour progression [95]. In this study, PD-L1 suppressor receptor was downregulated on DCs and this observation goes hand in hand with a decreased activation of Tregs and increased activation of effector T lymphocytes. All these changes might suggest that DCs are actively infiltrating tumours and aiding in the formation of antitumour immunity by activating effector T lymphocyte populations.

Additionally, increased expression levels of suppressor PD-L1 marker were observed on tumour-associated MDSCs, only in nsECT4-treated compared to untreated and μsECT-treated mice. In the literature, MDSCs are described as heterogeneous immature myeloid cells, capable of inhibiting lymphocytes and promoting rapid tumour growth [96,97,98]. Thus, this might explain the relation between increased PD-L1 on MDSCs and decreased tumour growth after nsECT4 treatment.

We observed no changes in the percentage of splenic monocytes. However, these monocytes were shown to have significantly higher expression levels of CD31 in most nsECT-treated mice. In a time-independent study, we observed that the percentage of splenic monocytes was normalised after the ECT treatment. A statistically significant decrease in the levels of adhesion molecule CD31 was observed on splenic monocytes, opposite to the results observed in time-dependent study. Some researchers [36,99,100] have shown that increased expression of CD31 enhance the migration of leukocytes. Thus, circulating CD31^+^ monocytes with high migration capacity can participate in angiogenesis of tumours. These monocytes can enter blood circulation and travel to the tumour microenvironment, depending on the stimuli, differentiate into TAMs and polarise towards the pro-inflammatory or anti-inflammatory phenotype. An increase of monocytes at the end of time-dependent study might relate to increased angiogenesis when tumours started regrowing. Furthermore, in the time-independent study, PD-L1 expression was decreased on CD31^+^ monocytes in lymph nodes of only nsECT4-treated mice. It suggests that these monocytes might be more active in the circulation and if they migrate to the tumour site, they could get less suppressed by the tumour microenvironment, possibly more differentiating into pro-inflammatory macrophages. Some researchers [101] have shown that proinflammatory TAMs are capable of normalising tumour vasculature, improving immune cell infiltration into tumours and increasing the efficiency of chemotherapy. However, another group of researchers [102] has shown that downregulated expression of CD31 on TAMs can upregulate the antibody-mediated phagocytosis of tumour cells. We observed that macrophages were increased in nsECT-treated mice. The ratio of pro-inflammatory to anti-inflammatory macrophage phenotypes has shown relatively increased pro-inflammatory TAMs in nsECT2, nsECT3 and nsECT4-treated mice. Furthermore, PD-L1 expression was decreased in pro-inflammatory TAMs in nsECT3 and μsECT-treated mice. Some studies [103,104] have shown that PD-L1 expression on macrophages downregulates phagocytosis and T cell stimulation. In this study, after nsECT treatment, increased splenic CD31^+^ monocytes possibly with enhanced migration capacity and increased transmigration to tissues could be related to an increase of macrophages in the tumours of ECT-treated mice. We suggest that after nsECT increased tumour-associated macrophages skew towards pro-inflammatory phenotype due to decreased PD-L1 levels and possibly phagocytose tumour cells, promoting antigen presentation following launched antitumour immunity.

Our described Lin^−^ population, as far as we know, is not yet present in the literature. In the time-dependent study, the percentage of this population was increased in spleens and tumours of nsECT2, nsECT3, nsECT4 and μsECT-treated compared to untreated tumour-bearing mice. However, in the time-independent study, we have not observed a significant increase of the Lin^−^ population after ECT treatment. The splenic Lin^−^ population was shown to express monocyte/macrophage marker CD163 [105], CD24, suppressor receptor PD-L1 [106,107,108], and antigen presentation molecules MHCII, CD80 and CD86. In time-independent study we have observed no significant differences in expression levels of PD-L1, CD24, MHCII and CD86 as it was noticed in a time-dependent study. Oppositely to the time-dependent study, the expression levels of CD40 were increased on this Lin^−^ population in lymph nodes and spleens of nsECT4-treated compared to untreated mice. According to the literature, CD86 and CD80 costimulatory molecules are known to be expressed on professional antigen presenting cells, stimulating the activation of T lymphocytes [109,110]. In cancer microenvironment, CD24 regulates cell migration, invasion and proliferation, also known as a biomarker of poor cancer prognosis [111]. However, in healthy organisms, CD24 is known as a co-activation molecule for the activation of T lymphocytes [112]. In our study, downregulated MHCII expression levels were observed on splenic Lin^−^ population in nsECT2, nsECT3 and μsECT-treated compared to untreated mice. Notably, the expression levels of MHCII were significantly higher on the splenic Lin^−^ population in nsECT4-treated compared to μsECT-treated mice. Downregulated expression of CD86 and upregulated expression of CD24 was observed in tumour-associated Lin^−^ population of most nsECT-treated mice. However, significantly downregulated expression of PD-L1 was observed on this Lin^−^ population in tumours of nsECT3 and nsECT4-treated compared to untreated mice. We propose that these cells might have a role in antigen presentation and activation of T lymphocytes in the tumour microenvironment, because of expressed MHCII, CD86 and CD80 markers. The expression of PD-L1 in nsECT3 and nsECT4-treated mice, possibly render this population to be more active in antitumour immune response. In order to identify if these Lin^−^ cells were innate lymphoid cells (ILCs), they were stained for CD127 and CD25. However, additional staining revealed that these cells are not ILCs (Figure S5). We further hypothesize if Lin^−^ population could be a subset of B cells [113]. According to this study, a subset of B cells that lacks CD19 and B220 expression, was reported to have a high expression of CD24 and MHCII. In our study, Lin^−^ population highly expressed both of these markers. Therefore, we suggest that this population might be implicated in antigen presentation, especially at the tumour site. Higher percentage of Lin^−^ cells observed in a time-dependent study after nsECT4 treatment might signify their role in repressing the growth of tumours. However, this Lin^−^ population might be highly heterogeneous and further studies need to be done to elucidate a precise role of these immune cells.

## 5. Conclusions

In conclusion, time-dependent and time-independent studies set up was used to assess mice survival, tumour growth and immune system dynamics in response to nsECT versus μsECT in different timepoints after treatment. A time-dependent study has shown that nsECT3 and nsECT4 treatment significantly prolonged the survival of C57BL/6J mice. Further, we assessed the differences of the effect between nanosecond and microsecond ECT on the immune system changes in time-dependent and time-independent in vivo studies. In both studies, the most prominent changes in immune cell populations were observed after nsECT4 compared to the μsECT treatment. 

Changes in lymphocyte subpopulations. This nanosecond treatment modality increased CD4^+^ T cells in all organs, splenic plasma, memory B cells and resulted in higher levels of antitumour IgG antibodies. Notably, in the time-independent study, nsECT4 resulted in more rapid production of antitumour antibodies in comparison to μsECT treatment. Remarkably, in the time-dependent study, nsECT4 treatment upregulated the PD-1 expression on splenic CD4^+^ Tr1 cells and downregulated GITR on splenic Tr1 cells and Tregs. The nsECT4 treatment resulted in increased splenic CD8^+^ T cells, whereas μsECT treatment resulted in decreased tumour-associated CD8^+^ T cells. Moreover, CD4^+^CD8^+^ T and plasma cells were also increased after nsECT4 treatment.

Changes in myeloid cell populations. In the time-independent study, an increased percentage of tumour-associated dendritic cells was observed. ECT treatment delayed an increase of splenic CD31^+^ monocytes that are implicated in angiogenesis and regrowing tumours. Time-dependent study has shown that increased CD31 expression on splenic monocytes could be linked to an increase of tumour-associated macrophages. Moreover, we observed a relatively increased proportion of tumour-associated pro-inflammatory macrophages. We also noticed that PD-L1 expression was decreased on tumour-associated DCs after ECT treatment, possibly modulating these DCs to be more active in antigen presentation. 

In both studies, we have described a Lin^−^ population that was increased in the spleens and tumour after ECT treatment in the time-dependent study. This Lin^−^ population was shown to express MHCII, CD80, CD86, macrophage marker CD163, suppressor markers PD-L1 and CD24. In time-dependent study, after nsECT4 treatment, MHCII did not decrease on splenic Lin^−^ population as compared to other treatment modalities. However, PD-L1 was significantly decreased on tumour-associated Lin^−^ population only after nsECT3 and nsECT4 treatments. All these immune system changes come hand in hand with increased survival rates in nsECT3 and nsECT4 treatment groups. Immune system dynamics observed in the time-dependent and time-independent studies hint fourth modality of nanosecond electrochemotherapy (nsECT4) to be more promising than standard μsECT (ESOPE) for the future development of combined anticancer treatment strategies.

## Figures and Tables

**Table 1:** Electroporation protocols employed in the study [8].

Protocol	Pulse Parameters	Energy (J)
nsPEF1	3.5 kV/cm × 200 ns × 200, 1 kHz	~0.5
nsPEF2	3.5 kV/cm × 200 ns × 200, 1 MHz	~0.5
nsPEF3	3.5 kV/cm × 700 ns × 200, 1 kHz	~1.6
nsPEF4	3.5 kV/cm × 700 ns × 200, 1 MHz	~1.6
μsPEF	1.3 kV/cm × 100 μs × 8, 1 Hz	~0.9

The pulse waveform and the spatial distribution of the electric field inside the tumour can be found in our previous study [8].

## Data Availability

Data is available from corresponding author AB on request.

## Supplementary Materials

The following are available online at https://www.mdpi.com/article/10.3390/cancers14246254/s1, Figure S1: Spleens of ECT-treated and untreated tumour-bearing mice; Figure S2: Common gating strategy to discriminate singlets, live and immune cells; Figure S3: Gating strategy of T and NKT cells; Figure S4: Gating strategy of myeloid cell subpopulations (monocytes/macrophages, granulocytes and dendritic cells); Figure S5: Gating strategy of Lin^−^ population; Figure S6: Gating strategy of B cell subsets; Figure S7: CD8^+^ T lymphocytes analysis in time-dependent study; Figure S8: CD8+ T lymphocytes analysis in time-independent study; Table S1: Antibodies used for the multicolour flow cytometry; Table S2: Expression of cell surface markers on Lin^−^ population in spleens; Table S3: Statistically significant changes in immune cell populations and their surface markers after ECT; Table S4: Statistically significant changes in immune cell surface markers after ECT.
