# Supplementary material for: High-Frequency Nanosecond Bleomycin Electrochemotherapy and its Effects on Changes in the Immune System and Survival
Source: Cancers (Basel). 2022 Dec 19;14(24):6254. doi: 10.3390/cancers14246254 (PMC9776811; doi:10.3390/cancers14246254)
Supplement: Supplementary file 1 [file cancers-14-06254-s001.zip › cancers-2048979-supplementary.pdf]

Supplementary data

Table S1. Antibodies used for the multicolour flow cytometry.

Anti-mouse antibodies against cell markers:	Conjugated fluorochromes	Abbreviation	Producers	Catalog#	Dilution
B220	PE	B220–PE	BD Biosciences, USA	553090	1:800
	PerCP	B220–PerCP		553093	1:200
	FITC	B220–FITC	BioLegend, USA	103205	1:800
CD11b (Mac1)	APC	CD11b–APC	Miltenyi Biotec GmbH, Germany	130-113-803	
	PE-TexasRed	CD11b–PE-TxRed	eBioscience, Invitrogen, Thermo Fisher Scientific, USA	RM2817	
CD11c	PE-TexasRed	CD11c–PE-TxRed		MCD11c17	
CD138	APC	CD138–APC		MA5-23553	1:200
CD163	PE	CD163–PE		12-1631-82	
CD19	eFluor506	CD19–eF506		69-0193-82	1:800
CD1d	eFluor450	CD1d–eF450		48-0011-82	
CD24	PerCP-eFluor710	CD24–PerCP-eF710		46-0242-80	
CD25	APC	CD25–APC		12-0251-81	1:200
CD27	Super Bright 600	CD27–SB600		63-0271-82	1:400
CD3e	FITC	CD3–FITC		11-0031-85	1:800
	eFluor450	CD3–eF450		48-0032-82	1:100
CD31	PE	CD31–PE	BD Biosciences, USA	553373	1:400
CD4	PerCP	CD4–PerCP		553052	1:800
	Alexa Fluor 647	CD4–AF647		557681	
	PE	CD4–PE	Miltenyi Biotec GmbH, Germany	130-091-607	
CD40	eFluor450	CD40–eF450	eBioscience, Invitrogen, Thermo Fisher Scientific, USA	48-0402-82	
CD43	PE	CD43–PE	Miltenyi Biotec GmbH, Germany	130-091-585	1:200

CD44	eFluor450	CD44–eF450	eBioscience, Invitrogen, Thermo Fisher Scientific, USA	48-0441-82	1:400
	FITC	CD44–FITC	BD Biosciences, USA	553133	1:800
	PE	CD44–PE		553134	1:400
CD5	PE	CD5–PE		553023	1:800
CD62L	APC	CD62L–APC	Miltenyi Biotec GmbH, Germany	130-091-805	1:200
CD80 (B7-1)	Super Bright 600	CD80–SB600	eBioscience, Invitrogen, Thermo Fisher Scientific, USA	63-0801-82	1:400
CD86 (B7-2)	FITC	CD86–FITC		11-0862-82	1:800
CD88 (C5aR)	Alexa Fluor 488	CD88–AF488	Bio-Rad Laboratories, USA	MCA2456A 488	
CD8	FITC	CD8–FITC	BD Biosciences, USA	553031	1:400
	PE	CD8–PE		553041	1:800
	Alexa Fluor 700	CD8–AF700	eBioscience, Invitrogen, Thermo Fisher Scientific, USA	56-0081-82	1:200
CD49b (DX5)	APC-Cy7	CD49b–APC-Cy7		A15420	1:400
F4/80	Alexa Fluor 647	F4/80–AF647		MF48021	
FR4	Brilliant Violet 421	FR4–BV421	BD Biosciences, USA	744119	
GITR (CD357)	Super Bright 600	GITR–SB600	eBioscience, Invitrogen, Thermo Fisher Scientific, USA	63-5874-82	1:800
Gr-1	PE	Gr-1–PE	BioLegend, USA	108407	
	FITC	Gr-1–FITC	Miltenyi Biotec GmbH, Germany	130-102-338	
MHCII	FITC	MHCII–FITC		130-123-666	1:50
PD-1 (CD279)	eFluor506	PD1–eF506	eBioscience, Invitrogen, Thermo Fisher Scientific, USA	69-9985-82	1:400
PD-L1 (CD274)	Super Bright 600	PD-L1–SB600		63-5982-82	1:800
Rat IgG1 kappa Isotype Control	Brilliant Violet 421	Iso–BV421	BD Biosciences, USA	562868	1:400
	Super Bright 600	Iso–SB600	eBioscience, Invitrogen, Thermo Fisher Scientific, USA	63-4031-30	1:400 – 1:800
Rat IgG2a kappa Isotype Control	PE	Iso–PE		12-4321-81	1:200 – 1:800
	eFluor506	Iso–eF506		69-4321-82	1:400 – 1:800

TCRγ/δ	PE	TCRγ/δ–PE	BD Biosciences, USA	553178	1:400
Ter119		Ter119–PE		553673	1:200

- Goat anti-mouse IgG AF488 (eBioscience, Invitrogen, Thermo Fisher Scientific, USA).
- Anti-mouse FcγR (Fc block) – supernatant from hybridoma cells secreting monoclonal antibodies against Fc receptor (home-made, CIM, Lithuania).
- Fluorescent stains: Yo-Pro-1, 0.1nM (Life Technologies, Thermo Fisher Scientific, USA); LIVE/DEAD™ Fixable Near-IR Stain (Thermo Fisher Scientific, USA).

Table S2. Expression of cell surface markers on Lin^{-} population in spleens.

**Receptors**	**Expression**	**Number of mice used for analysis**	**Function of the receptor**	
CD5	0–8 %	2	Suppressor function. Marker of T and B cells	
F4/80	4–8 %	2	Marker of murine macrophages	
CD44	5–7 %	2	Marker of at least once re-activated lymphocytes	
GITR	0–10 %	2	Marker of multiple times re-activated lymphocytes	
CD40	1–13 %	43	Marker of activated antigen presenting cells	
CD49b (DX5)	5–10 %	2	Marker of NK, NKT, Tr1 cells	
MHC II	5–45 %	43	Marker of antigen presenting cells	
CD80	10–50 %	43	Marker of antigen presenting cells (B cells, macrophages, and DCs). Costimulatory molecule for T-cell activation and regulation of the activity of normal and malignant B cells.	
PD-L1	20–77 %	43	Suppressor ligand expressed on APCs and B cells. Suppression of lymphocytes expressing PD-1 receptor.	
CD163	20–78 %	43	Polarization marker for pro-inflammatory macrophages	
CD86	30–85 %	43	Co-activation ('Signal 2') for antigen presentation	
CD24	42–98 %	43	This marker is expressed on hematopoietic and cancer cells. Can function as 'don't eat me' signal, inhibiting phagocytic of APCs like macrophages. Involved in discrimination between DAMP and PAMP signalling pathways. All functions are still unclear.	

Table S3. Statistically significant changes ($p < 0.05$) in immune cell populations and their surface markers after ECT, compared to untreated tumour-bearing mice.

	**Time-dependent study**		**Time-independent study**		
	In spleens	In tumours	In spleens	In tumours	In lymph nodes
All T cells	↓ nsECT1	–	↑ µsECT, nsECT4	–	–
CD4^{+}CD8^{+} (DP) T cells	↑ nsECT2/4	–	↓ nsECT4	–	↑ µsECT, nsECT4
CD4^{-}CD8^{-} (DN) T cells	–	–	↓ µsECT, nsECT4	–	–
Helper CD4^{+} T cells	↓ nsECT1	–	↑ µsECT, nsECT4	↑ nsECT4	↑ µsECT, nsECT4

CD4$^+$ Tr1 cells	↑ nsECT3	–	–	–	–
CD4$^+$ Treg	–	–	↓ nsECT4	–	↑ nsECT4
Cytotoxic CD8$^+$ T cells	↓ µsECT	–	↑ nsECT3	↓ µsECT	-
Memory CD8$^+$ T cells	*not assessed*	*not assessed*	–	↓ nsECT4	
CD4/CD8 ratio	–	↑	–	↑ µsECT, nsECT4	–
B cells	↓	↑ µsECT	–	–	↓ nsECT4
Memory B cells	–	*not assessed*	–	–	–
Plasma cells	↑ nsECT3/4	*not assessed*	–	–	–
NK cells	*not assessed*	*not assessed*	–	–	↑ µsECT, nsECT4
NKT cells	*not assessed*	*not assessed*	–	–	↑ µsECT, nsECT4
Monocytes / Macrophages	–	↑ nsECT2	–	–	–
CD11b$^+$ DC	–	–	–	–	↓ nsECT4
CD11b$^-$ DC	–	↓ nsECT2/4	–	–	–
CD163$^+$/ CD163$^-$ MΦ ratio	*not assessed*	↓ nsECT2	*not assessed*	*not assessed*	*not assessed*
"Negative" population	↑ nsECT1/2	–	↑	↓ µsECT	↓ µsECT, nsECT4

Table S4. Statistically significant changes ($p < 0.05$) in immune cell surface markers after ECT, compared to untreated tumour- bearing mice.

	**Time-dependent study**			**Time-independent study**		
Marker	**Immune cell populations**	**In spleens**	**In tumours**	**In spleens**	**In tumours**	**In lymph nodes**
GITR$^+$	CD4$^+$ T_{EM}	↓ nsECT2/4	–	–	–	–
	CD4$^+$ T_{Reg}	↓ µsECT, nsECT4	↑ nsECT3	–	–	–
	CD4$^+$ Tr1	↓ nsECT4	–	–	–	–
CD44+	Cytotoxic CD8$^+$ T cells	–	–	–	↓ nsECT4	–
	CD4$^+$ T_{CM}	–	↑	–	–	–
	CD4$^+$ T_{EM}	–	↑ nsECT2	–	–	–
	CD4$^+$ T_{Reg}	–	↑ nsECT2	–	–	–
CD40+	"Negative" population	–	–	↑ µsECT, nsECT4	–	↑ µsECT
	B cells	↓	–	–	–	–
PD-L1+	"Negative" population	–	↓ nsECT3/4	–	–	–
	CD163$^-$ MΦ ratio	–	↓ nsECT4	–	–	–
	CD11b$^-$ DC	–	↓ nsECT3	–	–	↓ nsECT4
	CD11b$^+$ DC	–	↓ nsECT4	↑ µsECT, nsECT4	–	–
	CD31$^+$ Monocytes	–	–	–	–	↓ nsECT4
	MDSC	–	–	–	↑ nsECT4	–
CD86+	"Negative" population	–	↓	–	↑ nsECT4	–
CD24+	"Negative" population	–	↑ nsECT2/3/4	–	–	–
MHCII	"Negative" population	↓ nsECT2/3, µsECT	–	–	–	–
CD31+	CD11b$^+$ DC	–	–	–	–	↓ µsECT
	Monocytes	↑ nsECT3/4, µsECT	–	–	–	–
Ter119+	Erythrocytes	*not assessed*	*not assessed*	↓ µsECT, nsECT4	*not assessed*	*not assessed*

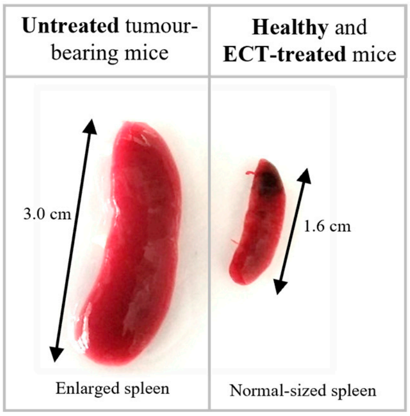

Figure S1. Spleens of ECT-treated and untreated tumour-bearing mice.

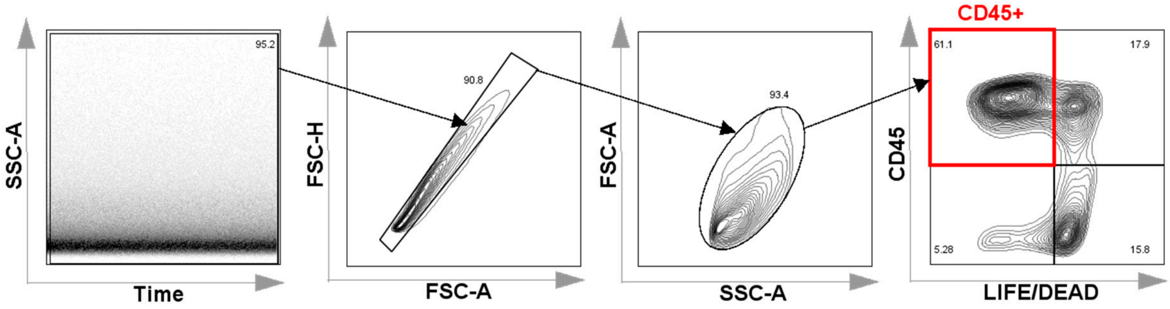

Figure S2. Common gating strategy to discriminate singlets, live and immune cells.

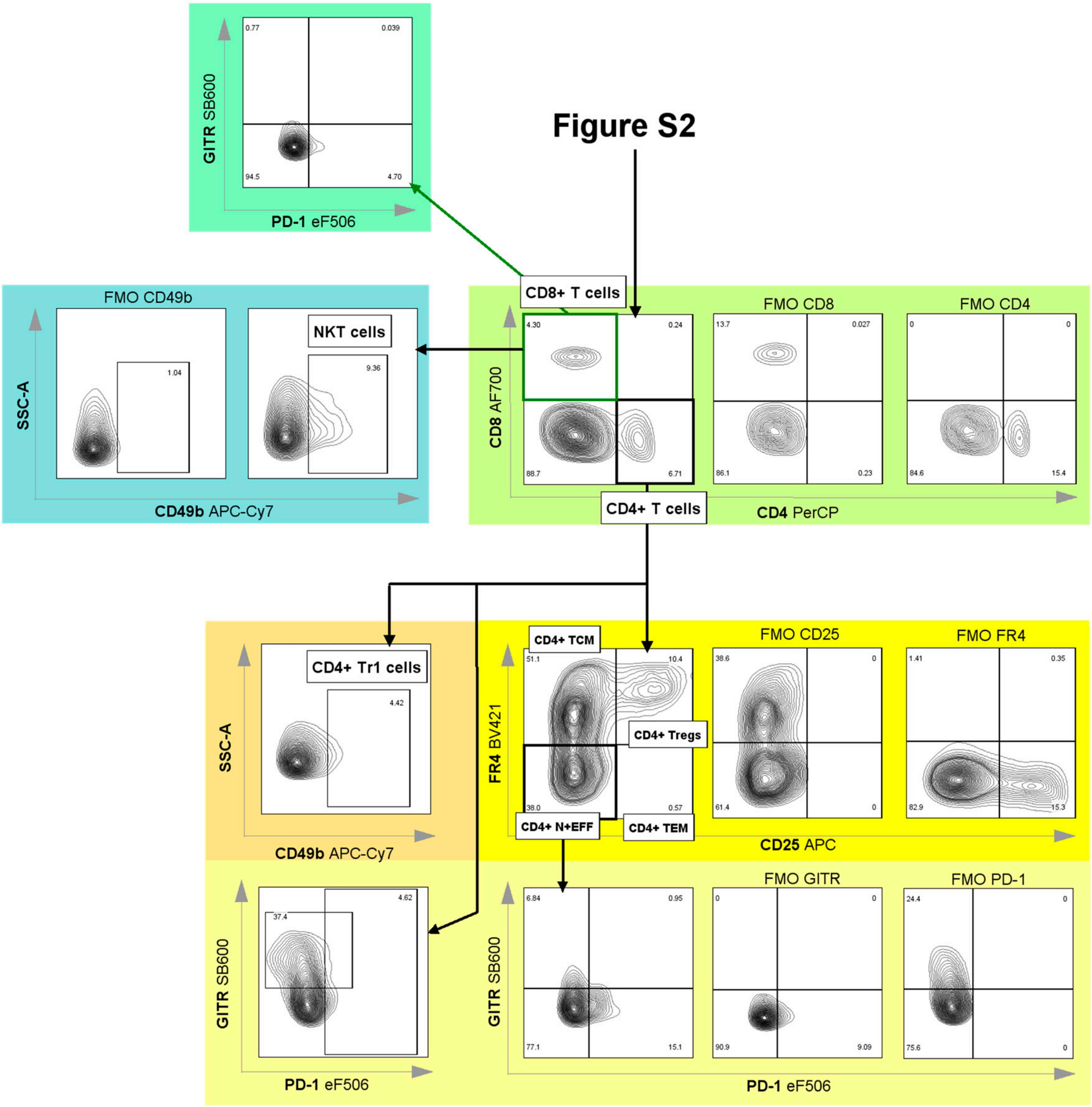

Figure S3. Gating strategy of T and NKT cells.

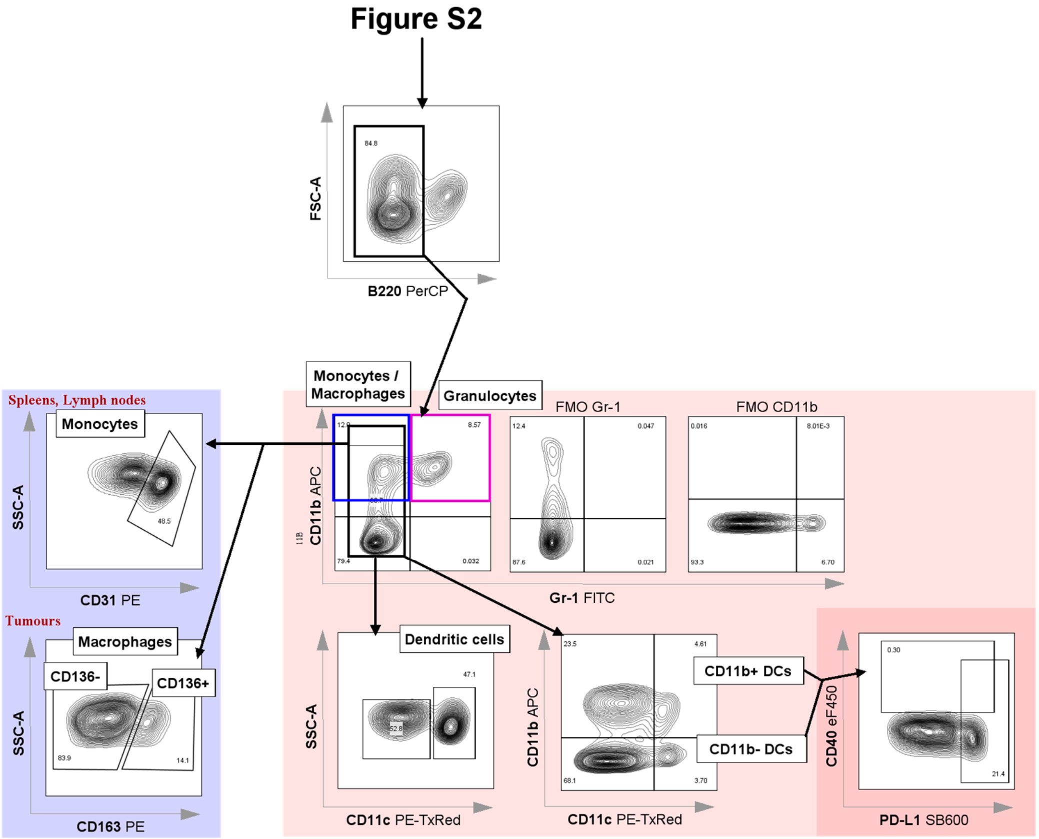

Figure S4. Gating strategy of myeloid cell subpopulations (monocytes/macrophages, granulocytes and dendritic cells).

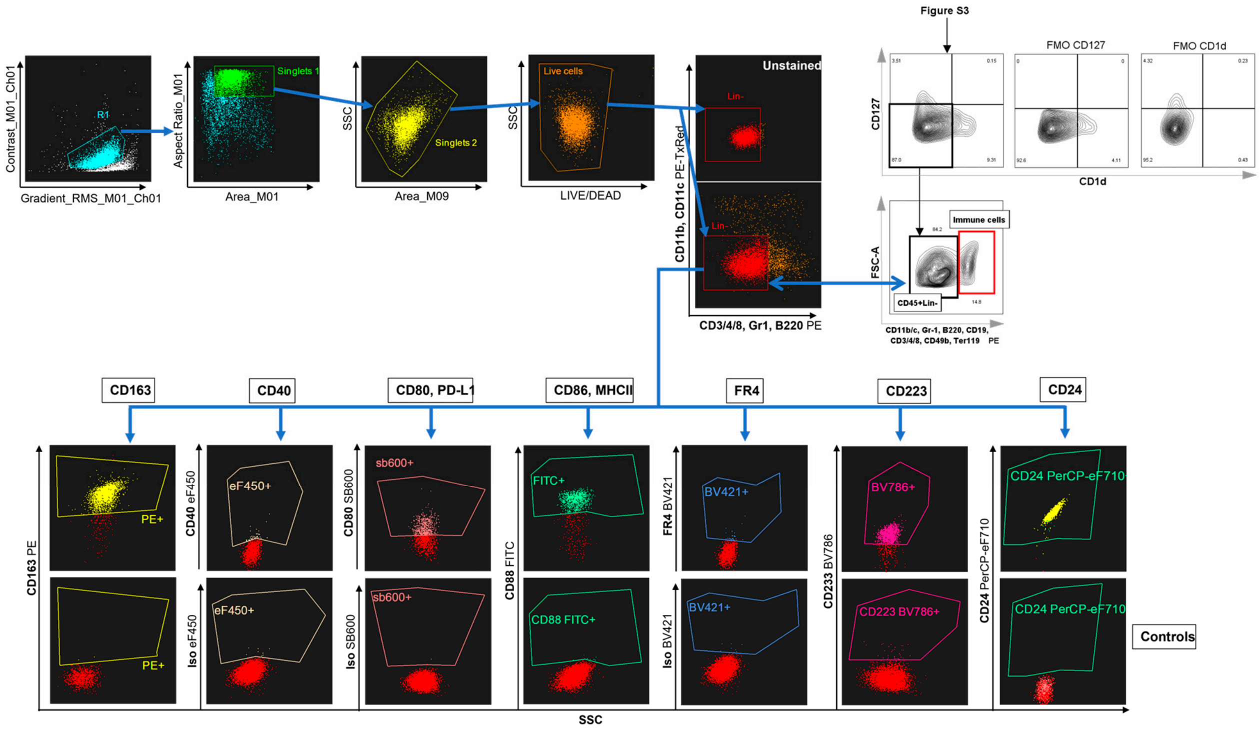

Figure S5. Gating strategy of Lin⁻ population.

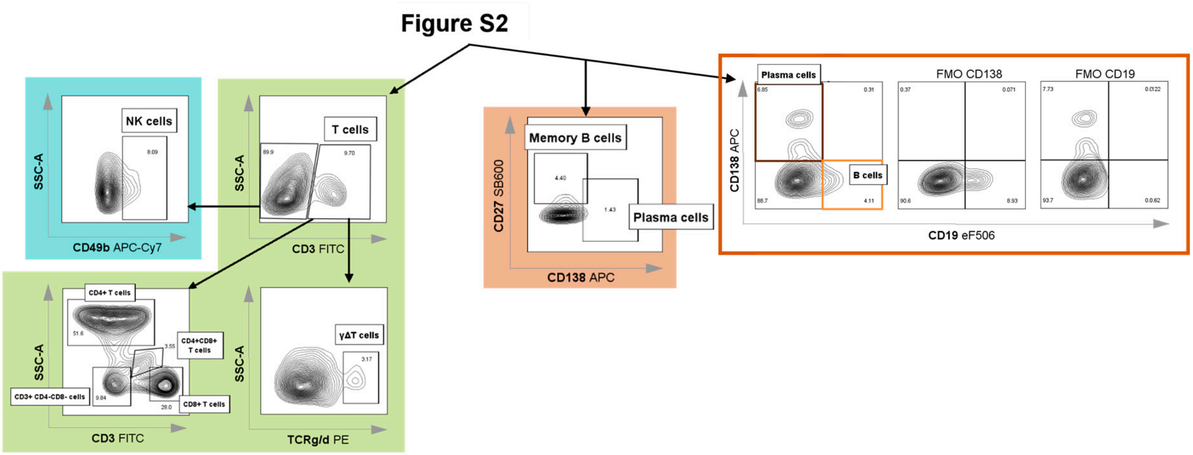

Figure S6. Gating strategy of B cell subsets.

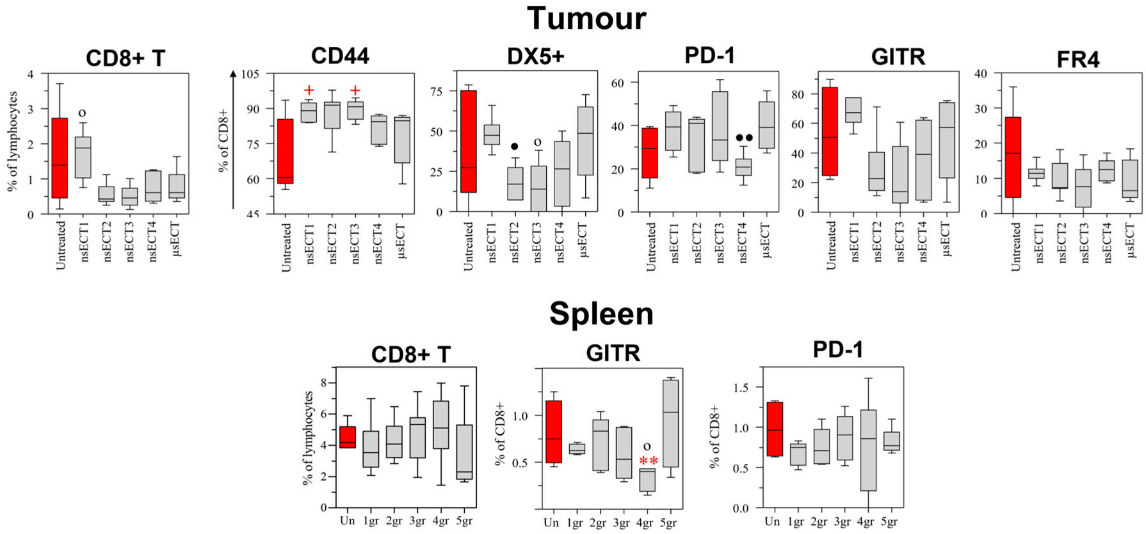

Figure S7. CD8⁺ T lymphocytes analysis in time-dependent study. Cytometry was performed with BD FACSAria III cytometer. Statistically significant (** $p < 0.005$) differences and tendencies (+ p = 0.05–0.1) compared to the untreated group. Significant differences (● $p < 0.05$; ●● $p < 0.005$) and tendencies (o p = 0.05–0.1) compared to µsECT group.

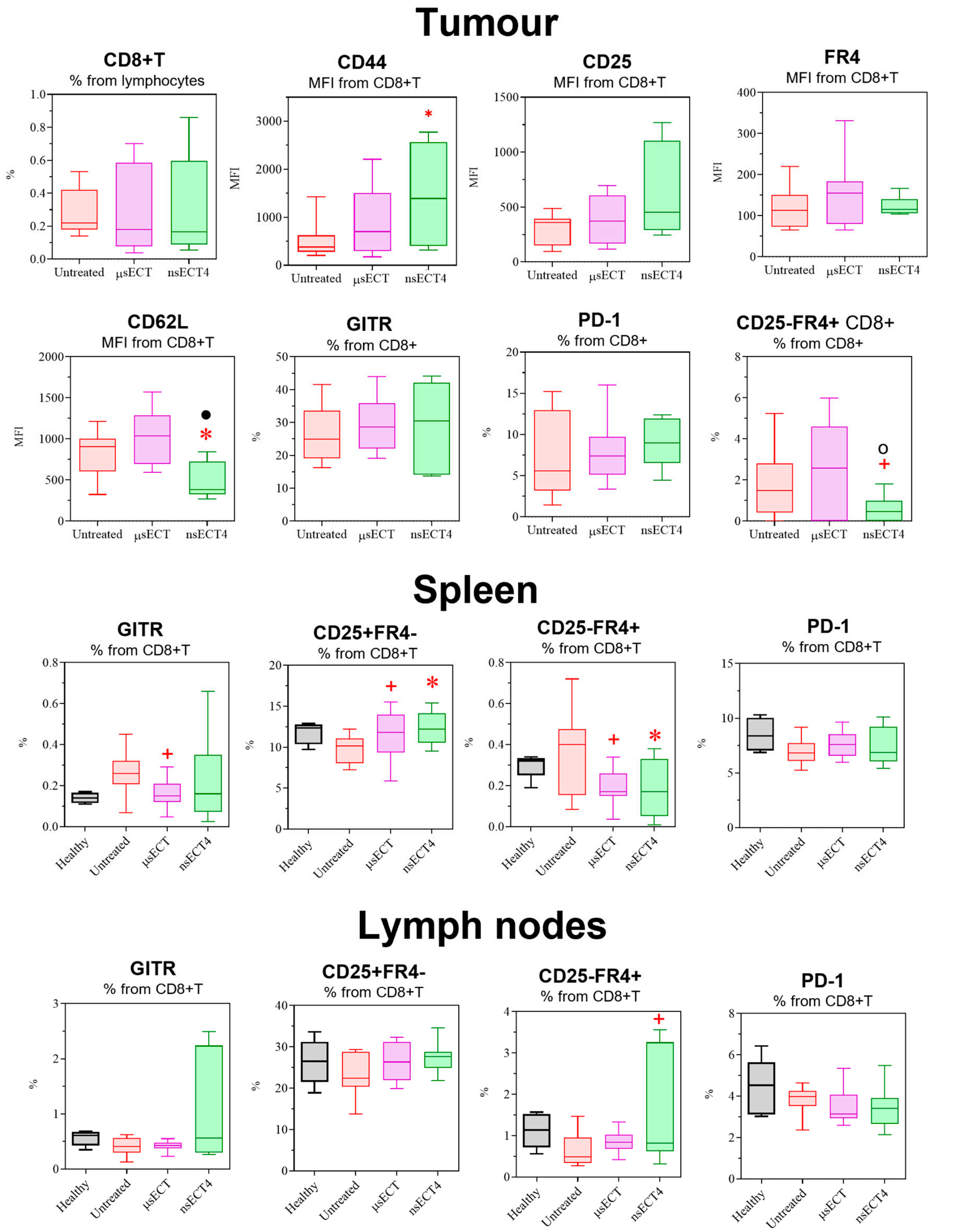

Figure S8. CD8⁺ T lymphocytes analysis in time-independent study. Cytometry was performed with BD FACSAria III cytometer. Statistically significant (* $p < 0.05$) differences and tendencies (+ p = 0.05 – 0.1) compared to the untreated group. Significant differences (● $p < 0.05$) and tendencies (o p = 0.05–0.1) compared to µsECT group.
